# NPINN+: an enhanced physics-informed neural network for solving wave equations with nonlocal boundary conditions

**DOI:** 10.1038/s41598-026-50374-9

**Published:** 2026-05-19

**Authors:** Qiancheng Tan, Shuyun Yang, Yonghui Qin

**Affiliations:** 1 College of Mathematics and Computing Science, Guangxi Colleges and Universities Key Laboratory of Data Analysis and Computation, Guilin University of Electronic Technology, Guilin, 541004 China; 2 Center for Applied Mathematics of Guangxi, Guilin University of Electronic Technology, Guilin, 541004 China; 3 Guangxi Key Laboratory of Automatic Detecting Technology and Instruments, Guilin University of Electronic Technology, Guilin, 541004 China

**Keywords:** Physics-informed neural networks (PINN), Nonlocal condition, Wave equation, Numerical simulation, Dynamic softAdapt loss weighting, Mathematics and computing, Physics

## Abstract

Wave equations with nonlocal conditions appear in many scientific and engineering applications, such as, the population dynamics, the mathematical biology, and the materials science. The numerical challenge mainly stems from nonlocal terms, whose global coupling degrades the efficiency and stability of classical methods. In recent years, physics-informed neural networks (PINN) have achieved notable success in solving partial differential equations. In this paper, we propose an enhanced physics-informed neural network for wave equations subject to nonlocal conditions, termed NPINN+. By exploiting an equivalent transformation of the nonlocal condition, the original problem is reformulated into a wave equation satisfying Neumann boundary conditions with an additional integral-form source term. NPINN+ employs a single neural network to provide a unified representation of the spatiotemporal solution, while incorporating the governing equation, derivative information, initial and boundary conditions, and nonlocal constraints into a unified physics-informed loss function, enabling effective capture of the underlying physical features. Furthermore, a residual-based dynamic sampling strategy and a SoftAdapt-driven adaptive loss weighting mechanism are introduced to enhance accuracy and training robustness. Numerical experiments on regular domains demonstrate the effectiveness of the proposed method, and its extension to star-shaped domains is achieved via a polar coordinate transformation. Comparative results with PINN, APINN, and RAR-PINN show that NPINN+ consistently achieves superior accuracy and stability.

## Introduction

The wave equations with the nonlocal conditions are widely applied in various scientific fields^1–6^, such as plasma physics, solid state physics, and fluid dynamics. In the mathematical theory research of partial differential equations, the nonlocal condition problems are different from the traditional local boundary condition ones, such as, Dirichlet boundary conditions, Neumann boundary conditions, and Robin boundary conditions^7–9^, namely, the behavior of a solution at a point on the boundary depends not only on the local properties at that point but also on the solution’s values over a broader region or the entire domain. As is well known the wave equations with nonlocal conditions are generally more difficult to solve the exact solution and analyze than their local counterparts as pointed out in^10–14^. Therefore, some traditional numerical methods have been developed for solving the partial differential models subject to a non-local conservation condition, such as finite difference methods^15,16^, finite element methods^17,14,18^, and spectral element methods^19–21^. However, for solving high-dimensional problems, these methods are often inefficient due to their high demands on storage and computational time. This is particularly evident in numerical approaches for mathematical physics equations on complex domains, such as the finite element method and spectral element methods, which typically depend on constructing suitable mesh partitions and applying mesh refinement to enhance solution accuracy^22,23^. Nowadays, a deep learning framework based on physics-informed neural networks (PINNs) was proposed for solving forward and inverse problems involving nonlinear partial differential equations (PDEs) in^24^. A primary advantage of PINNs is that the underlying physics is integrated into the loss function to guide the learning process, ensuring that predictions adhere to the governing equations^25,24^ without explicit domain discretization^26^. This approach improves accuracy and reduces reliance on large training datasets^27^. Subsequently, various advancements have been developed, such as adversarial uncertainty quantification^28^, physics-informed genetic programming for PDE discovery^29^, and applications of PINNs in peridynamic models^30^, spherical domains^31^, and domain decomposition^32^. Moreover, PINNs have shown great potential in hemodynamics, where variable separated architectures^33^ and integral conservation laws^34^ have been integrated to simulate 3D blood flow in complex arterial geometries. Furthermore, transfer learning and invariant deep neural networks have been explored to solve inverse problems and complex PDEs^35,36^. Despite these developments, the application of PINNs to models with non-local boundary conditions remains relatively unexplored due to training instability and sensitivity to hyperparameters^37,38^. To address these issues, various strategies such as adaptive loss weighting, adaptive activation functions^34^, and dynamic sampling have been proposed to enhance the convergence and accuracy of PINNs^39–41,33^.

In this paper, we consider an initial boundary value problem for the wave equations as follows

1 $$\begin{aligned} \left\{ \begin{array}{lllll} \partial _{t}^2 V(\boldsymbol{x},t)-c^2\Delta V=Q(\boldsymbol{x},t), & \quad (\boldsymbol{x},t)\in \Omega \times (0,T], \\ V(\boldsymbol{x},0)=V_0(\boldsymbol{x}), \quad \partial _{t}V(\boldsymbol{x},0)=V_1(\boldsymbol{x}),& \quad \boldsymbol{x}\in \Omega , \end{array}\right. \end{aligned}$$

where $\Omega \subset \mathbb R^d(d=1,2,3)$ denotes the physical space and its boundary is denoted by $\partial \Omega$, and $\boldsymbol{x}=x$ for $d=1$, $\boldsymbol{x}=(x_1,x_2)$ for $d=2$, and $\boldsymbol{x}=(x_1,x_2,x_3)$ for $d=3$, the boundary condition of (1) is given as

$$\begin{aligned} V|_{\boldsymbol{x}\in \partial \Omega }=0,&\quad 0<t\le T, \end{aligned}$$

and $V(\boldsymbol{x},t):\Omega \times (0,T]\rightarrow \mathbb R$ is the solution of (1), which satisfies the nonlocal condition

2 $$\begin{aligned} \int _{\Omega } V(\boldsymbol{x},t)d\boldsymbol{x}=E(t),\quad 0<t\le T. \end{aligned}$$

Assume that the right-hand side function $Q(\boldsymbol{x},t):\Omega \times (0,T]\rightarrow \mathbb R$ and the initial values $V_0(\boldsymbol{x}), V_1(\boldsymbol{x}):\Omega \rightarrow \mathbb R$ are smooth functions, and the coefficient *c* is a given constants.

**Figure 1:**
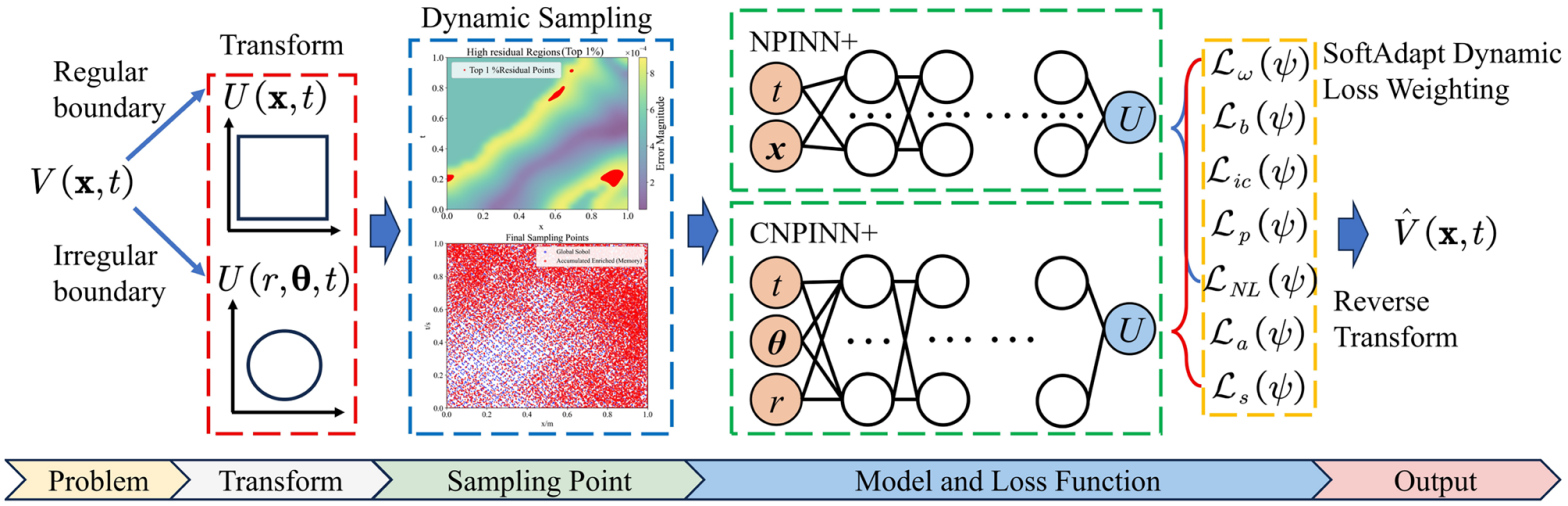
Illustration of a NPINN+ with Coordinate-Transformed.

As we all know, wave equations with nonlocal boundary conditions is that matrices of corresponding operators are not symmetric, which is as opposed to the classical boundary conditions^42–44^. At the same time, it is a challenge to construct an effective numerical method for solving the equations with nonlocal boundary conditions due to the effects of non-local memory^45,46^. In this paper, we investigate the PINN deep-learning approach based on the adaptive loss weighting and dynamic sampling for wave equations (1) with the nonlocal conditions (2). The architecture diagram of our method is shown as in Figure 1. The proposed method transforms the original wave equation with satisfying nonlocal conditions into an equivalent wave-type integral equation on a regular physical space, which include the bounded interval, the rectangular region, and the hexahedral region. Numerical examples are applied to test the effectiveness of our method. By the coordinate transformation, the proposed method is effective for learning the wave equation (1) with the nonlocal conditions (2) on the circular domain and the pentagram region, respectively. Our contributions of this work are summarized as follows

- The SoftAdapt method is employed to dynamically adjust loss weights, significantly accelerating convergence, with noticeable improvement observed within 5000 iterations.
- A hybrid strategy combining Sobol sequences with dynamic sampling reduces the error to nearly half.
- A tailored loss function is proposed for wave equations with nonlocal condition, validated on 1D, 2D, and 3D cases, achieving a maximum error on the order of $10^{-3}$.

The paper is organized as follows: Section 2 introduces the wave equations with nonlocal constraints and reformulates them into a standard form with integral source terms. We then describe the NPINN with Coordinate-Transformed+ model, including its composite loss, dynamic sampling, and SoftAdapt-based weighting. Section 3 presents numerical results on 1D, 2D, and 3D wave problems with passive and active sources, demonstrating the model’s accuracy and efficiency. Section 4 concludes the paper and outlines future work on nonlinear extensions and architecture optimization.

## Methodology

In this section, we present the NPINN+ framework for solving the wave equation (1) with the nonlocal condition (2). The method incorporates adaptive loss weighting and dynamic sampling to enhance training stability and accuracy. In the PINN architecture, the space time variables $(\boldsymbol{x}, t)$ serve as network inputs, enabling automatic differentiation to compute all required derivatives with machine precision, in contrast to finite-difference approximations that become unreliable for deep networks. Leveraging this capability, NPINN+ embeds the governing equation, boundary conditions, and nonlocal constraints into a unified loss formulation, and efficiently solves the resulting reformulated wave-integral system across one-, two-, and three-dimensional domains.

### Wave equations with the nonlocal conditions

Now, without loss of generality, we present an equivalent system of wave equations (1) with the nonlocal condition (2) for $E(t)=0$. For simplicity, we consider the spatial domain $\Omega := (0, l)^d$ for the wave equations in (1).

By integrating (1) with respect to $\boldsymbol{x}$ over (0, *l*), we obtain

$$\begin{aligned} \int _{0}^l \partial _{x}^2V(x,t)dx = \partial _{x}V(0, t)-\partial _{x}V(l, t)=\int _{0}^{l} Q(x, t) d x \end{aligned}$$

for $$d=1$$;

$$\begin{aligned}&\quad -\int _{\Omega }\nabla \cdot \nabla V d\Omega =-\oint _{\partial \Omega }\nabla V\cdot \textbf{n} d s \\ &=\int _{0}^l\partial _{x_1} V(0,x_2,t)dx_2 +\int _{0}^l\partial _{x_2} V(x_1,0,t)dx_1 -\int _{0}^l\partial _{x_1} V(l,x_2,t)dx_2 -\int _{0}^l\partial _{x_2} V(x_1,l,t)dx_1 =\int _{\Omega } Q(\boldsymbol{x}, t) d \Omega \end{aligned}$$

for $$d=2$$;

$$\begin{aligned}&\quad -\int _{\Omega }\nabla \cdot \nabla V =-\oint _{\partial \Omega }\nabla V\cdot \textbf{n} d S \\ &=\int _{0}^l\int _{0}^l\partial _{x_1} V(0,x_2,x_3,t)-\partial _{x_1} V(l,x_2,x_3,t)dx_2dx_3 +\int _{0}^l\int _{0}^l\partial _{x_2}V(x_1,0,x_2,t)-\partial _{x_2}V(x_1,l,x_2,t)dx_1dx_3 \\ &\qquad +\int _{0}^l\int _0^l\partial _{x_3}V(x_1,x_2,0,t)-\partial _{x_3} V(x_1,x_2,l,t)dx_1dx_2 =\int _{\Omega } Q(\boldsymbol{x}, t) d \Omega \end{aligned}$$

for $d=3$, where *ds* and *dS* are the element of arc length and curved surface on the boundary $\Omega$, respectively.

Let us introduce a new unknown function

3 $$\begin{aligned} U(\boldsymbol{x}, t)=V(\boldsymbol{x}, t)+\frac{1}{(2 l)^d}\sum _{s=1}^dx_s^{2} \int _{\Omega } Q(\boldsymbol{x}, t) d \Omega . \end{aligned}$$

Therefore, Problems (1) is equivalent to the following equations

4 $$\begin{aligned} \left\{ \begin{array}{lllll} \frac{\partial ^{2} U}{\partial t^{2}}-\Delta U=P(\boldsymbol{x}, t), & \quad (\boldsymbol{x},t)\in (0,l)^d\times (0,T], \\ U(\boldsymbol{x}, 0)=\omega (\boldsymbol{x}), \quad \partial _{t}U(\boldsymbol{x}, 0)=\gamma (\boldsymbol{x}), & \quad \boldsymbol{x}\in \Omega , \\ U(\boldsymbol{x}, t)|_{x_s=0 }=0,\quad [\nabla U(\boldsymbol{x},t)\cdot \textbf{n}]_{x_s=0}=[\nabla U(\boldsymbol{x},t)\cdot \textbf{n}]_{x_s=l}, & \quad t\in (0, T], \quad s\le d, \end{array}\right. \end{aligned}$$

where

5 $$\begin{aligned}&P(\boldsymbol{x},t)=Q(\boldsymbol{x},t) +\frac{1}{(2 l)^d}\sum _{s=1}^dx_s^{2} \int _{\Omega } \partial _{t}^2Q(\boldsymbol{x},t)d\Omega -\frac{1}{l^d}\int _{\Omega } Q(\boldsymbol{x},t)d\Omega , \end{aligned}$$

6 $$\begin{aligned}&\omega (\boldsymbol{x})=V_0(\boldsymbol{x})+\frac{1}{(2 l)^d}\sum _{s=1}^dx_s^{2} \int _{\Omega } Q(\boldsymbol{x},0)d\Omega , \quad \gamma (\boldsymbol{x})=V_1(\boldsymbol{x})+\frac{1}{(2 l)^d}\sum _{s=1}^dx_s^{2} \int _{\Omega } \partial _{t}Q(\boldsymbol{x},0)d\Omega . \end{aligned}$$

### PINN for the wave equation with the nonlocal condition

**Figure 2:**
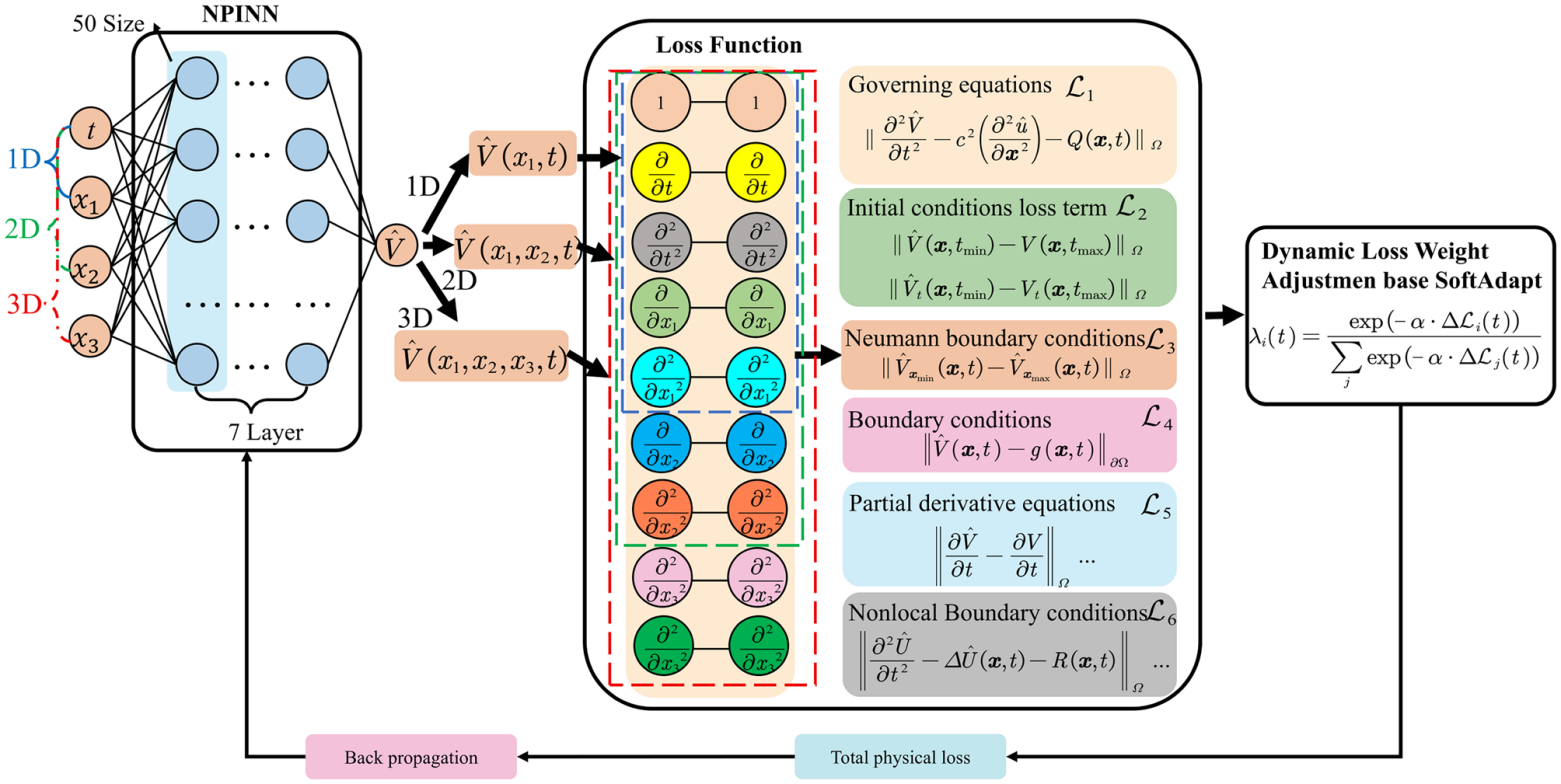
NPINN structure for one- ,two- and three-dimensional wave equation problem.

Here, we present a simple description of the PINN for the wave equations (1) with nonlocal condition as present in Figure 2.

Let

$${\boldsymbol{y}} = \mathcal{N}\mathcal{N}(\boldsymbol{x}; \textbf{W}, \textbf{b}) : \mathbb {R}^{d_{\boldsymbol{x}}} \rightarrow \mathbb {R}^{d_{\boldsymbol{y}}}$$

be an *L*-layer feed-forward neural network, where $\boldsymbol{x}$ and $\boldsymbol{y}$ denote the input data and the output data, respectively. We denote the network depth by *L*. For the forward propagation, we set $\boldsymbol{z}^0 \equiv \boldsymbol{x}$ and $\boldsymbol{z}^L \equiv \boldsymbol{y}$.

7 $$\begin{aligned} \boldsymbol{z}^{l}=\sigma ^{l}\left( \textbf{W}^{l} \cdot \boldsymbol{z}^{l-1}+\textbf{b}^{l}\right) , \quad l=1, \ldots , { L}, \end{aligned}$$

where $\sigma ^l$ denotes the activation functions, $\textbf{W}^l, \textbf{b}^l$ are the parameters of the *l*-th layer, which are called the weights and biases, respectively. Next, we first give the Artificial Neural Networks (ANN) with ${\mathbb K}$ hidden layers and taking the activation function for all layers $\sigma ^l=\tanh (\cdot )$ except the last as follows

$$\begin{aligned} \begin{aligned}&\boldsymbol{z}^{1} = \tanh \left( \textbf{W}^{1} \cdot \boldsymbol{x}+ \textbf{b}^{1}\right) ,\\&\boldsymbol{z}^{l+1} = \tanh \left( \textbf{W}^{l+1} \cdot \boldsymbol{z}^{l} + \textbf{b}^{l+1}\right) , \\&\textbf{u} = \textbf{W}^{l+2} \cdot \boldsymbol{z}^{l+1} + \textbf{b}^{l+2}, \qquad l=1,\cdots ,{ L}. \end{aligned} \end{aligned}$$

Next, we describe a residual-based dynamic sampling strategy integrated with the Sobol sequence to enhance the training of NPINN. In this approach, loss weights are dynamically adjusted according to the magnitude of the residuals, balancing the contributions of different loss terms and promoting convergence. This strategy follows the adaptive weighting method introduced in^47^. The Sobol sequence, on the other hand, is a low-discrepancy quasi-random sequence constructed via base-2 expansions and direction numbers^48,49^, which facilitates efficient space-filling during sampling.

**Figure 3:**
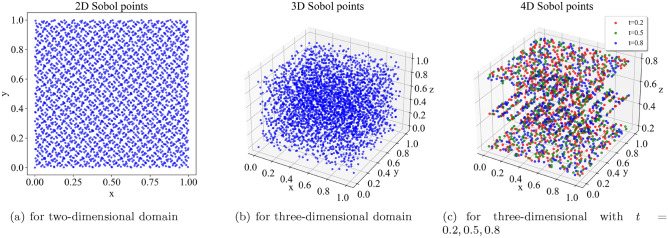
The point distributions generating by the Sobol sequence in different domain.

The sample points are generated within $(0, l)^d$ (for example, with $L=1$ as illustrated in Figure 3) using a Sobol sequence. This method generates points deterministically so that each new point fills the gaps between existing ones in a near-optimal manner, thereby improving sampling efficiency even in moderately high dimensions.

The *i*-th point of the sequence in the *j*-th dimension is computed using direction numbers and a bitwise exclusive-or (XOR) operation ^50^:

$$x_{i}^{(j)} = \bigoplus _{k=0}^{L} a_k^{(j)} v_k^{(j)},$$

where $v_k^{(j)}$ are the direction numbers specific to dimension *j*, $a_k^{(j)}$ are the binary digits of the index *i*, and $\oplus$ denotes the bitwise XOR operation.

Figure 3(a) shows the distribution of points in a two-dimensional spatial domain at a fixed time *t*. The three-dimensional case at a fixed *t* is displayed in Figure 3(b), while Figure 3(c) presents the point distributions for three different time instances, $t=0.2, 0.5, 0.8$.

**Algorithm 1:**
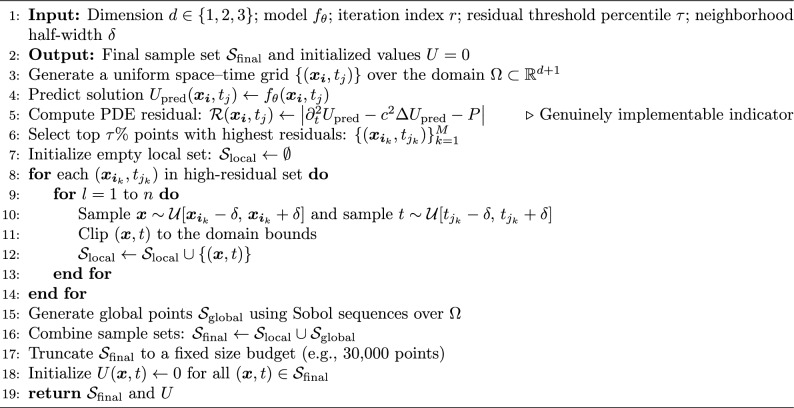
Residual-Driven Adaptive Dynamic Sampling Algorithm for NPINN+ in *d* Spatial Dimensions

Now, we describe the dynamic loss weighting strategy based on the SoftAdapt technique ^51^. Herein, we give a simple description of composite loss function for the NPNNs method. Based on the automatic differentiation method^52^, we apply the partial derivatives $\partial _t U$, $\partial _{x_s}U$, $\partial _{t}^2 U$, and $\partial _{x_s}^2 U$ $(s \le d)$ to define the composite loss function. Explicitly, it takes the form

8 $$\begin{aligned} \mathcal {L}(\psi )=\mathcal {L}_w(\psi ) +\mathcal {L}_{b}(\psi )+\mathcal {L}_{ic}(\psi ) +\mathcal {L}_{p}(\psi )+\mathcal {L}_{NL}(\psi ), \end{aligned}$$

where $\psi$ denotes the trainable parameters of the neural network. Here, $\mathcal {L}_w$ is defined by the governing wave equation, $\mathcal {L}_{b}$ corresponds to the boundary conditions, $\mathcal {L}_{ic}$ is obtained from the initial conditions, and $\mathcal {L}_{p}$ relates to the partial derivative matching. Notably, the term $\mathcal {L}_{NL}$ enforces the nonlocal condition, which is newly introduced in this work. This overall formulation follows the classical PINN loss design proposed by Raissi et al.^24^. Next, let $\mathcal{N}\mathcal{N}(\boldsymbol{x}, t; \psi )$ be the training solution obtained by the NPINN model ( here abbreviated as $\mathcal{N}\mathcal{N}$ ) to predict the displacement $u(\boldsymbol{x}, t)$. Here, five components of (8) are defined as

$$\begin{aligned}&\mathcal {L}_{w}(\psi ):= \frac{1}{N_w}\sum _{i=1}^{N_w} \Big | \partial _{t}^2 \mathcal{N}\mathcal{N}(\boldsymbol{x}_i,t_i;\psi ) - c^2 \Delta \mathcal{N}\mathcal{N}(\boldsymbol{x}_i,t_i;\psi ) - P(\boldsymbol{x}_i,t_i) \Big |^2, \\&\mathcal {L}_{ic}(\psi ):= \frac{1}{N_{ic}}\sum _{i=1}^{N_{ic}} \Big ( \big | \mathcal{N}\mathcal{N}(\boldsymbol{x}_i,0;\psi )-\omega (\boldsymbol{x}_i)\big |^2 + \big | \partial _t \mathcal{N}\mathcal{N}(\boldsymbol{x}_i,0;\psi )-\gamma (\boldsymbol{x}_i)\big |^2 \Big ), \\&\mathcal {L}_{\text {b}}(\psi ):= \frac{1}{N_d} \sum _{i=1}^{N_d} \sum _{s=1}^d \Big | \big [ \mathcal{N}\mathcal{N}(\boldsymbol{x}, t_i; \psi )-\mathcal {B}_D(\boldsymbol{x}, t_i)\big ]_{x_s=0} \Big |^2 \\ &\qquad \qquad + \frac{1}{N_n} \sum _{i=1}^{N_n} \sum _{s=1}^{d} \Big | \big [ \textbf{n}_j \cdot \nabla _{\boldsymbol{x}} \mathcal{N}\mathcal{N}(\boldsymbol{x}, t_i; \psi )\big ]_{x_s=0} - \big [ \textbf{n}_j \cdot \nabla _{\boldsymbol{x}} \mathcal{N}\mathcal{N}(\boldsymbol{x}, t_j; \psi )\big ]_{x_s=l} \Big |^2, \\&\mathcal {L}_p(\psi ):= \frac{1}{N_p}\sum _{i=1}^{N_p} \Big ( \big | \mathcal{N}\mathcal{N}_t(\boldsymbol{x}_i,t_i;\psi ) - U_t(\boldsymbol{x}_i,t_i) \big | + \sum _{s=1}^{d}\big [ \big | \mathcal{N}\mathcal{N}_{x_s}(\boldsymbol{x}_i,t_i;\psi ) - U_{x_s}(\boldsymbol{x}_i,t_i)\big | \\&\qquad \qquad + \big | \mathcal{N}\mathcal{N}_{tt}(\boldsymbol{x}_i,t_i;\psi ) - U_{tt}(\boldsymbol{x}_i,t_i)\big |^2 + \big | \mathcal{N}\mathcal{N}_{x_s t}(\boldsymbol{x}_i,t_i;\psi ) - U_{x_st}(\boldsymbol{x}_i,t_i)\big |^2 \\&\qquad \qquad + \big | \mathcal{N}\mathcal{N}_{x_s x_s}(\boldsymbol{x}_i,t_i;\psi ) - U_{x_sx_s}(\boldsymbol{x}_i,t_i)\big |^2 \big ] \Big ), \\&\mathcal {L}_{NL}(\psi ):= \frac{1}{N_w} \sum _{i=1}^{N_w} \Big | \partial _{t}^2 \mathcal{N}\mathcal{N}(\boldsymbol{x}_i,t_i; \psi ) - c^2 \Delta U(\boldsymbol{x}_i,t_i; \psi ) - R(\boldsymbol{x}_i,t_i) \Big |^2, \end{aligned}$$

where $$\boldsymbol{x}_{\boldsymbol{i}}:=(\xi _{i_1},\cdots ,\xi _{i_d}),~~ {\boldsymbol{i}}:=(i_1,\cdots , i_d),~~ 0\le i_s\le N$$, and

$$\begin{aligned}&\mathcal {B}_D(\boldsymbol{x}, t):= U_D(t) - \frac{1}{(2l)^d}\sum _{s=1}^d x_s^2 \int _{\Omega } Q_D(t)\, d\Omega , \\&R(\boldsymbol{x}, t):= P(\boldsymbol{x}, t) - \Big ( Q(\boldsymbol{x}, t) + \frac{1}{(2l)^d}\sum _{s=1}^d x_s^{2} \int _\Omega \partial _t^2 Q(\boldsymbol{x}, t)\, d\Omega - \frac{1}{l^d} \int _0^l Q(\boldsymbol{x}, t)\, d\Omega \Big ) . \end{aligned}$$

We define the index set $\mathbb {K}:= \{w, bc, ic, p, NL\}$, and let $\mathcal {L}_\kappa (e)$ denote the value of the $\kappa$-th loss component at training epoch *e*, where $\kappa \in \mathbb {K}$. The relative change of each loss term is computed as:

$$\Delta _\kappa (e) = \mathcal {L}_\kappa (e-1) - \mathcal {L}_\kappa (e), \quad \kappa \in {\mathbb K}$$

where *e* is the training epoch, and $\Delta _\kappa (e)$ reflects the decrease in the $\kappa$-th loss component from epoch $e-1$ to *e*. SoftAdapt calculates the dynamic weight for each loss component using a softmax-like function

$$\omega _\kappa (e) = \frac{\exp (-\alpha \, \Delta _\kappa (e))}{\sum _{j\in \mathbb {K}} \exp (-\alpha \, \Delta _j(e))},$$

where $\alpha > 0$ is a temperature parameter controlling the sharpness of the weighting distribution. This mechanism assigns larger weights to losses that decrease more slowly, thereby directing the model’s focus towards more challenging objectives. The total weighted loss at iteration *e* is then given by

$$\mathcal {L}_{\textrm{total}}(e) = \sum _{\kappa \in {\mathbb K}} \omega _\kappa (e) \, \mathcal {L}_\kappa (e).$$

Next, in order to balance each loss component dynamically during training and avoid any single term dominating, we apply a weight adjustment strategy as in Algorithm 2. This adaptive weighting scheme improves training stability and promotes balanced convergence by assigning dynamic weights $\omega _\kappa (e)$ to each loss component at epoch *e*, where *e* denotes the training epoch index and $\omega _\kappa (e)$ reflects the inverse rate of decrease in the corresponding loss term, as computed by the SoftAdapt formula. Specifically, a loss component that decreases more slowly (i.e., has smaller $\Delta _\kappa (e)$) will receive a relatively larger weight $\omega _\kappa (e)$, encouraging the model to focus on harder-to-fit terms 4.

**Algorithm 2:**
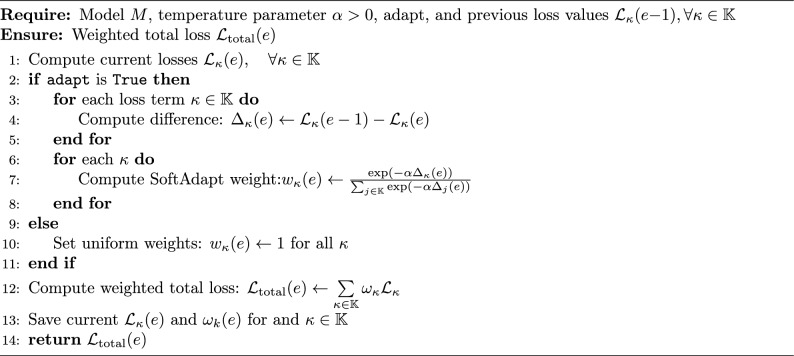
NPINN+ with SoftAdapt dynamic loss weighting

The NPINN+ framework employs a ”discovery-and-resolution” loop where residual-based dynamic sampling identifies high-error regions and populates them with new collocation points. Simultaneously, the SoftAdapt mechanism monitors PDE loss spikes and dynamically upscales weights $\omega _\kappa$ to force the optimizer to resolve intricate physical features and sharp gradients. This integrated synergy ensures high-precision capture of complex geometric features through adaptive spatial and weight optimization.

**Figure 4:**
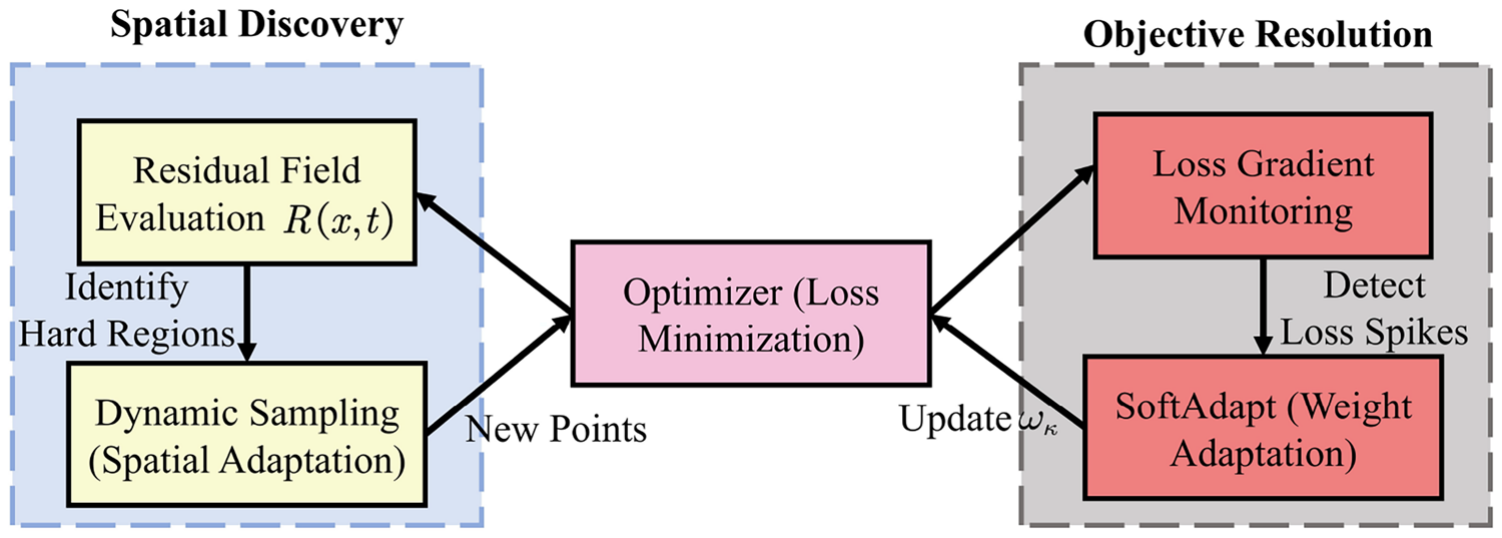
The synergy mechanism of the NPINN+ framework, illustrating the closed-loop interaction between spatial discovery and objective resolution.

## Numerical examples

This section presents numerical experiments to evaluate the performance of the proposed method. For simplicity, Let

$$\begin{aligned} \mathcal {E}(\boldsymbol{x},t):=(V_\textrm{pred}- V_\textrm{ref})(\boldsymbol{x}, t). \end{aligned}$$

Thus, the relative $L^2$-error $\mathcal {R}(\boldsymbol{x},t)$, the mean absolute error (MAE) $\mathcal {\bar{E}}(\boldsymbol{x},t)$, and the mean squares error (MSE) $\mathcal {E}_2(\boldsymbol{x},t)$ are defined as

9 $$\begin{aligned} \mathcal {R}(t) = \frac{\sqrt{\sum \limits _{\boldsymbol{i}=1}^N |\mathcal {E}({\boldsymbol{x_i}}, t) |^2}}{\sqrt{\sum \limits _{\boldsymbol{i}=1}^N | V_{\textrm{ref}}({\boldsymbol{x_i}}, t) |^2}}, \quad \mathcal {\bar{E}} (\boldsymbol{x},t):= \frac{1}{N} \sum _{\boldsymbol{i}=1}^N \left| \mathcal {E}({\boldsymbol{x_i}}, t) \right| \quad \mathcal {E}_2(\boldsymbol{x},t) = \frac{1}{N} \sum _{\boldsymbol{i}=1}^N \mathcal {E}^2({\boldsymbol{x_i}}, t). \end{aligned}$$

where $V_\textrm{pred}$ is obtained from $U_\textrm{pred}$ by applying the inverse transform corresponding to (3).

In the following 1D wave equation examples, we employ 12, 000 sampling points to train the $\mathcal {L}_{w}$ and $\mathcal {L}_{p}$ terms of the loss function, while using 3, 000 sampling nodes for $\mathcal {L}_b$ and $\mathcal {L}_{ic}$. For the 2D cases in Examples 3, 4 and 6 , we correspondingly adopt 25, 000 points for $\mathcal {L}_{w}$ and $\mathcal {L}_{p}$, and 6, 000 points for $\mathcal {L}_b$ and $\mathcal {L}_{ic}$. In Example 5, the values are set to 30, 000 for $\mathcal {L}_{w}$ and $\mathcal {L}_{p}$, and 9, 000 for $\mathcal {L}_b$ and $\mathcal {L}_{ic}$.

To ensure reproducibility, the NPINN+ framework is implemented in **Python 3.9.13 (PyTorch 1.10.0, CUDA 11.3)** on a workstation with an **Intel i5-14600KF** CPU and **NVIDIA RTX 4060 Ti** GPU. All stochastic components are fixed with a random seed of **1234**. Training employs a two-stage strategy: first, the **Adam** optimizer ($lr=10^{-3}$, **StepLR** with $\gamma =0.9$, step=1000) stabilizes the loss; subsequently, the **L-BFGS** optimizer (strong_wolfe line search, max 50,000 iterations) ensures high-precision convergence. Network weights are initialized via **Xavier initialization** with the $\tanh$ activation function, as detailed in Table 1.

**Table 1:** Hyperparameter settings and training configurations.

**Optimization**	**Detail**	**Model Setting**	**Value**
Stage 1 (Adam)	$lr=10^{-3}$, StepLR	Activation	$\tanh$
Stage 2 (L-BFGS)	Max iter: 50,000	Initialization	Xavier
Line Search	strong_wolfe	Random Seed	1234
Framework	PyTorch 1.10.0	Environment	RTX 4060 Ti

### For 1D wave equations

#### Example 1

Consider the wave equations (1) with (2) and

$$\begin{aligned}&Q(x, t)=0, \quad V_0(x)=\cos (\pi x), \quad V_1(x)=0, \quad V(0,t)=s(t)=\cos (\pi t), \quad \int _{0}^1V(x,t)dx=E(t)=0, \end{aligned}$$

where $(x,t)\in (0,1)\times (0, T]$. Assume that the exact solution is $V\left( x,t \right) ={\frac{1}{2}\Big [ \cos \left( \pi \left( x+t \right) \right) +\cos \left( \pi \left( x-t \right) \right) \Big ]}.$

In this example 1, we test the accuracy and effectiveness of our method for training 1D wave equations with $E(t)=0$. The following experiments demonstrate the effects of the absolute-error-driven adaptive dynamic sampling algorithm and the SoftAdapt dynamic loss weighting strategy, as well as their impact on the overall performance of the NPINN+ method.

**Table 2:** The MAE and training time for NPINN with different hidden layer depths (with slight random variation).

Hidden layer depth	$M=50$		$M=100$		$M=200$	
	MAE	Time(s)	MAE	Time(s)	MAE	Time(s)
1	$3.20\times 10^{-3}$	85	$2.75\times 10^{-3}$	142	$2.30\times 10^{-3}$	305
2	$2.65\times 10^{-3}$	110	$2.10\times 10^{-3}$	218	$1.75\times 10^{-3}$	525
3	$2.10\times 10^{-3}$	136	$1.55\times 10^{-3}$	388	$1.10\times 10^{-3}$	925
4	$1.60\times 10^{-3}$	160	$1.10\times 10^{-3}$	690	$8.50\times 10^{-4}$	1380
5	$1.25\times 10^{-3}$	190	$8.00\times 10^{-4}$	1120	$6.50\times 10^{-4}$	1980
6	$1.00\times 10^{-3}$	225	$6.20\times 10^{-4}$	1680	$5.34\times 10^{-4}$	2750
7	$1.35\times 10^{-3}$	252	$8.10\times 10^{-4}$	2260	$7.20\times 10^{-4}$	4380
8	$1.55\times 10^{-3}$	285	$9.20\times 10^{-4}$	3150	$8.10\times 10^{-4}$	6020

We first discuss the network architecture of the NPINN+. To achieve a balance between predictive accuracy and computational cost, a neural network with 7 layers and a width of $M=100$ is selected as the optimal configuration. As shown in Table 2, increasing either the network depth or width generally leads to a reduction in the mean absolute error (MAE), but at the expense of significantly longer training times. The chosen architecture attains a near-optimal MAE while substantially reducing the computational cost compared with deeper or wider networks (e.g., 8 layers with $M=200$), thereby providing an effective trade-off between accuracy and efficiency. The comprehensive experimental setup—including the Adam learning rate, inverse time decay scheduler, Xavier initialization, and hardware configuration—is summarized in Table 1; furthermore, the “estimated perturbations” in Table 2 are calculated as the standard deviation across 10 independent runs with fixed random seeds (0–9) to ensure reproducibility.

**Figure 5:**
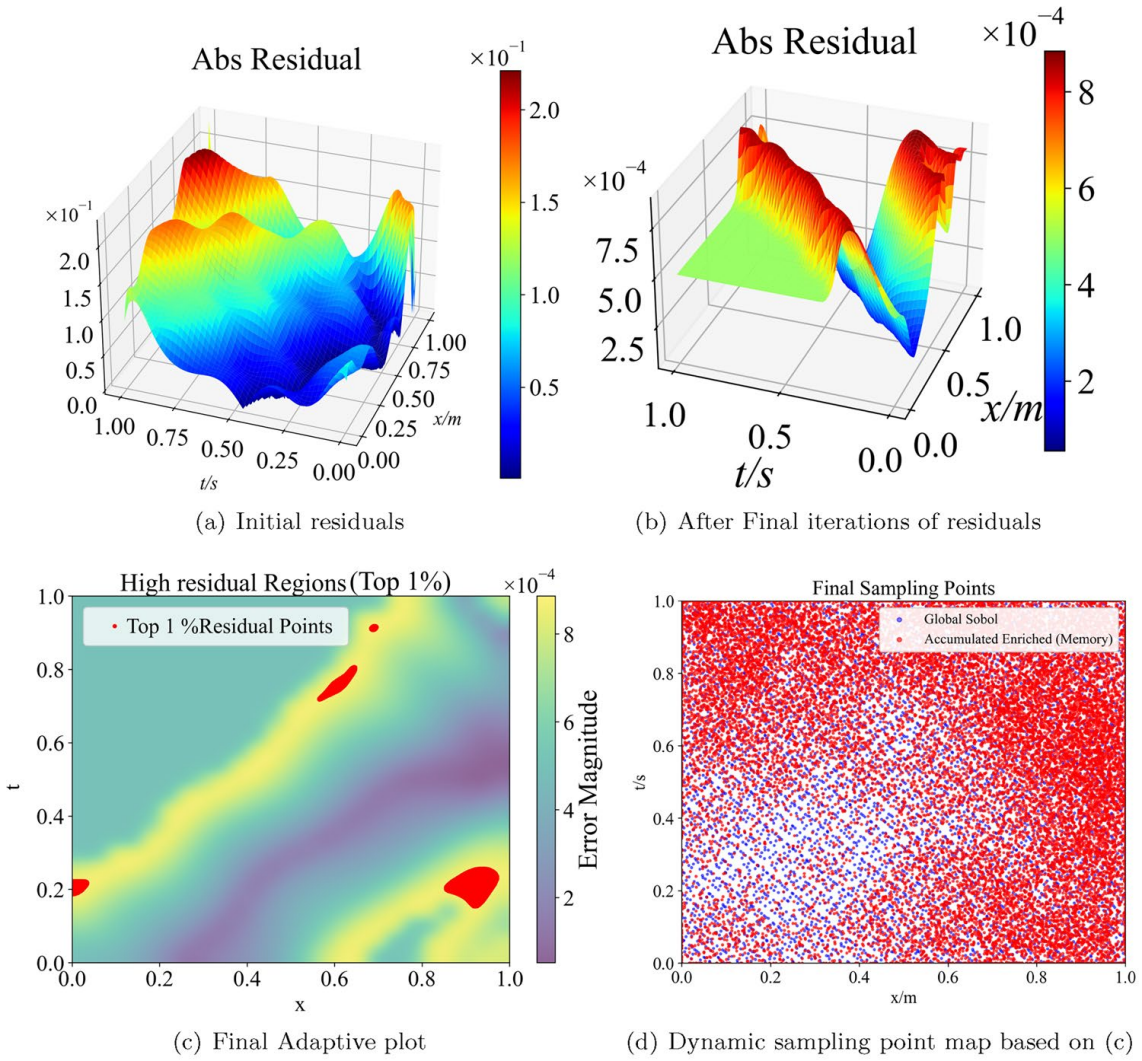
Comparison plot of adaptive dynamic sampling.

Here, the Sobol sequence algorithm is first employed to generate the initial collocation points, and numerical results reveal pronounced boundary errors, as shown in Figure 5(a), indicating a clear spatial non-uniformity in the error distribution. To address this issue, a residual-based adaptive dynamic sampling strategy is introduced to refine the sampling distribution by reallocating collocation points toward regions with larger residuals (see Algorithm 1).

Prior to the main computations, a preliminary experiment was conducted to validate the proposed adaptive dynamic sampling strategy and to determine an appropriate number of collocation points. Taking Example 1 as an illustration, the error threshold was set to $\tau =0.1$, meaning that the top $0.1\%$ of collocation points with the largest residuals were selected for refinement at each iteration. Since the total number of sampling points in the testing stage was 10, 000, an additional 1, 000 points were introduced per iteration, while the model was trained for a fixed 1, 000 epochs per iteration. As the adaptive sampling process proceeded, the overall error decreased steadily from $3\times 10^{-2}$ and stabilized after approximately three iterations at a level of about $4.0\times 10^{-4}$, corresponding to roughly 4, 000 collocation points. This result indicates that the proposed method is capable of achieving a favorable balance between accuracy and computational efficiency with a relatively small number of collocation points, as summarized in Table 3.

**Table 3:** Iterative NPINN training results with estimated perturbations (MAE, Relative $L^2$ Error, MSE, and Time).

Points	MAE	Relative $L^2$ Error	MSE	Time (s)
1000	$3.12(\pm 0.27) \times 10^{-3}$	$3.45(\pm 0.31)\times 10^{-3}$	$1.21(\pm 0.11)\times 10^{-5}$	$20.3(\pm 1.1)$
2000	$1.17(\pm 0.11) \times 10^{-3}$	$1.25(\pm 0.10)\times 10^{-3}$	$1.42(\pm 0.12)\times 10^{-6}$	$20.1(\pm 1.3)$
5000	$9.03(\pm 0.47) \times 10^{-4}$	$9.12(\pm 0.39)\times 10^{-4}$	$4.72(\pm 0.15)\times 10^{-7}$	$25.2(\pm 1.5)$
10000	$5.12(\pm 0.53) \times 10^{-4}$	$5.20(\pm 0.41)\times 10^{-4}$	$2.05(\pm 0.16)\times 10^{-7}$	$30.1(\pm 2.0)$
12000	$4.16(\pm 0.53) \times 10^{-4}$	$4.23(\pm 0.41)\times 10^{-4}$	$2.05(\pm 0.16)\times 10^{-7}$	$32.1(\pm 2.0)$
15000	$6.97(\pm 0.49) \times 10^{-4}$	$7.05(\pm 0.44)\times 10^{-4}$	$2.45(\pm 0.18)\times 10^{-7}$	$34.3(\pm 2.4)$
30000	$4.08(\pm 0.51) \times 10^{-4}$	$4.15(\pm 0.40)\times 10^{-4}$	$1.75(\pm 0.15)\times 10^{-7}$	$48.0(\pm 3.0)$

**Figure 6:**
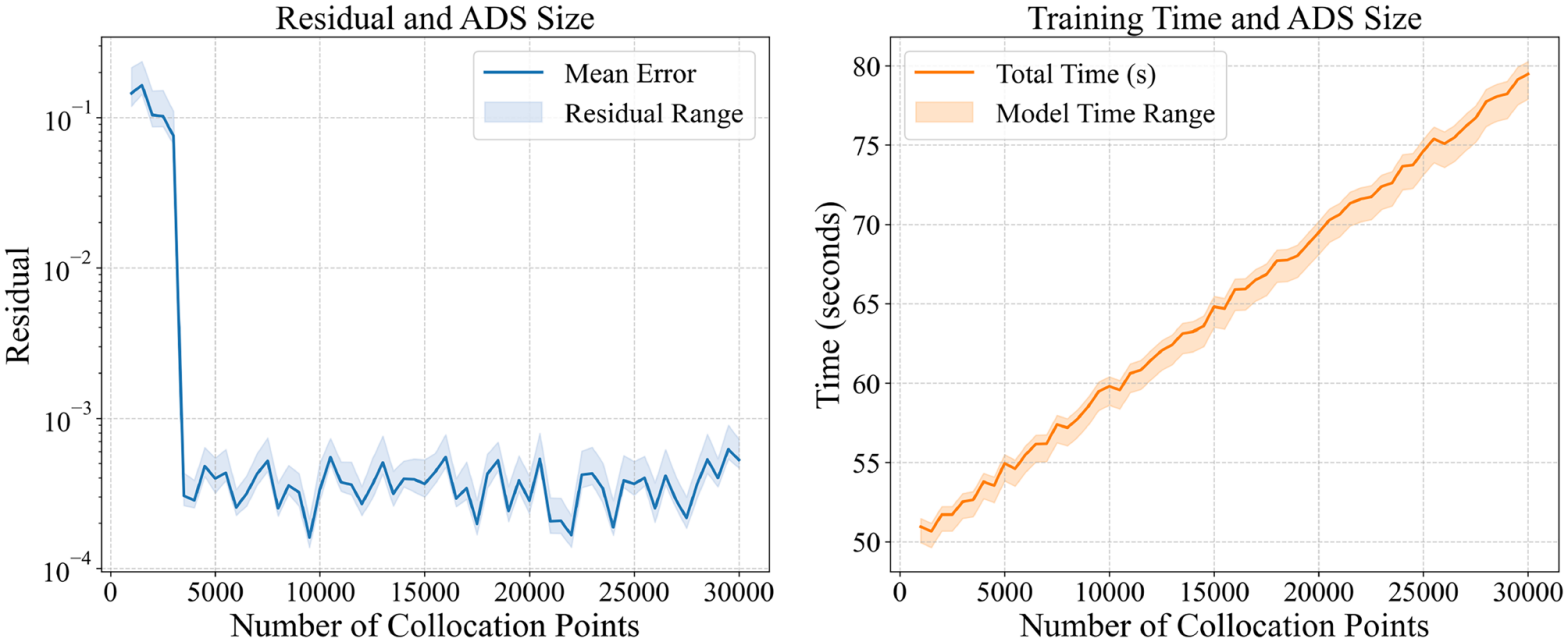
Training error and computational time across iterations using adaptive dynamic sampling.

Beyond this stage, further increases in the number of collocation points yield only marginal improvements in accuracy, while the computational cost continues to rise, with each iteration requiring approximately 30 seconds of training time. The error reduction process is illustrated in Fig. 5(a) and (b), and the spatial distribution of high-error regions together with the adaptively selected sampling points is shown in Fig. 5(c) and (d). The evolution of the error over 80 iterations, presented in Fig. 6, further confirms the convergence and stabilization behavior. Nevertheless, to reduce potential sampling bias and to provide a denser and more reliable representation of the solution space, the adaptive sampling procedure was continued in subsequent experiments, and the total number of collocation points was ultimately increased to approximately 12, 000, thereby enhancing the robustness and reliability of the final numerical results. The same experimental procedure is adopted for all subsequent examples and will not be repeated for brevity.

**Figure 7:**
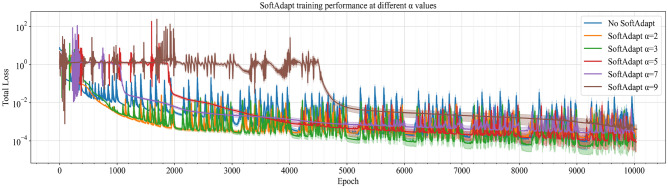
SoftAdapt training performance at different $\alpha$ values.

Finally, we investigate the influence of different values of $\alpha$ in the SoftAdapt dynamic loss weighting strategy. By promoting a balanced optimization among all physical constraint terms, this strategy effectively enhances both the convergence behavior and generalization capability of the model. As shown in Fig. 7, when $\alpha = 3$, the model exhibits a faster loss decay and a more efficient training process. Therefore, $\alpha = 3$ is adopted as the default setting in all subsequent numerical experiments.

The spatio-temporal sampling points of the PDE residual loss term, initial condition loss term, boundary condition loss term, and customized loss function are generated using the Sobol sequence algorithm, respectively, $N_p=12000$ (PDE residual points), $N_d=3000$ (initial condition points), and $N_n=3000$ (boundary condition points), using a fully-connected neural network with 7 hidden layers and 100 neurons per layer.

**Figure 8:**
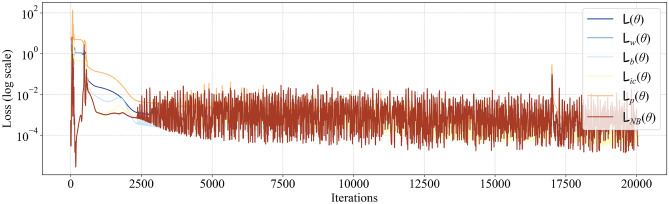
Evolution of different loss components during training in Example 1 (passive terms).

Next, Example 1 is used to assess the effectiveness of the proposed NPINN+ model in solving one-dimensional integral equations with a passive term. The model is trained using the Adam optimizer for 20,000 iterations. Figure 8 illustrates the convergence behavior of the loss components together with the relative error.

**Figure 9:**
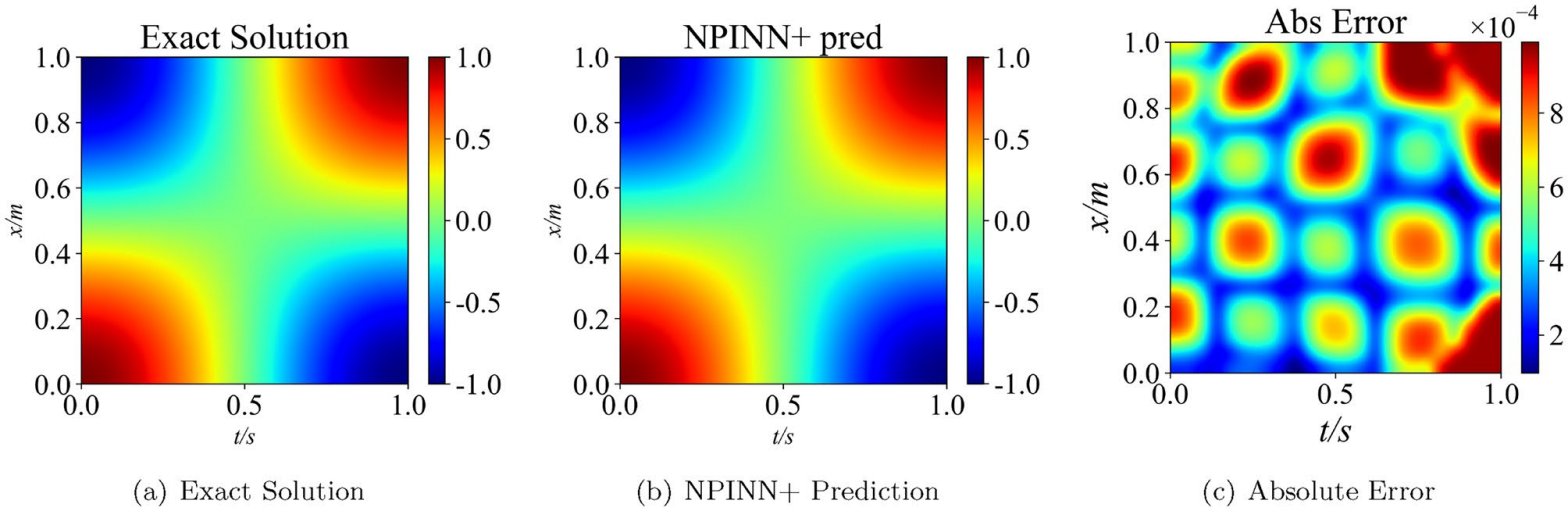
Comparison of NPINN+ predictions and exact solutions for 1D wave equations (passive terms, Example 1).

**Figure 10:**
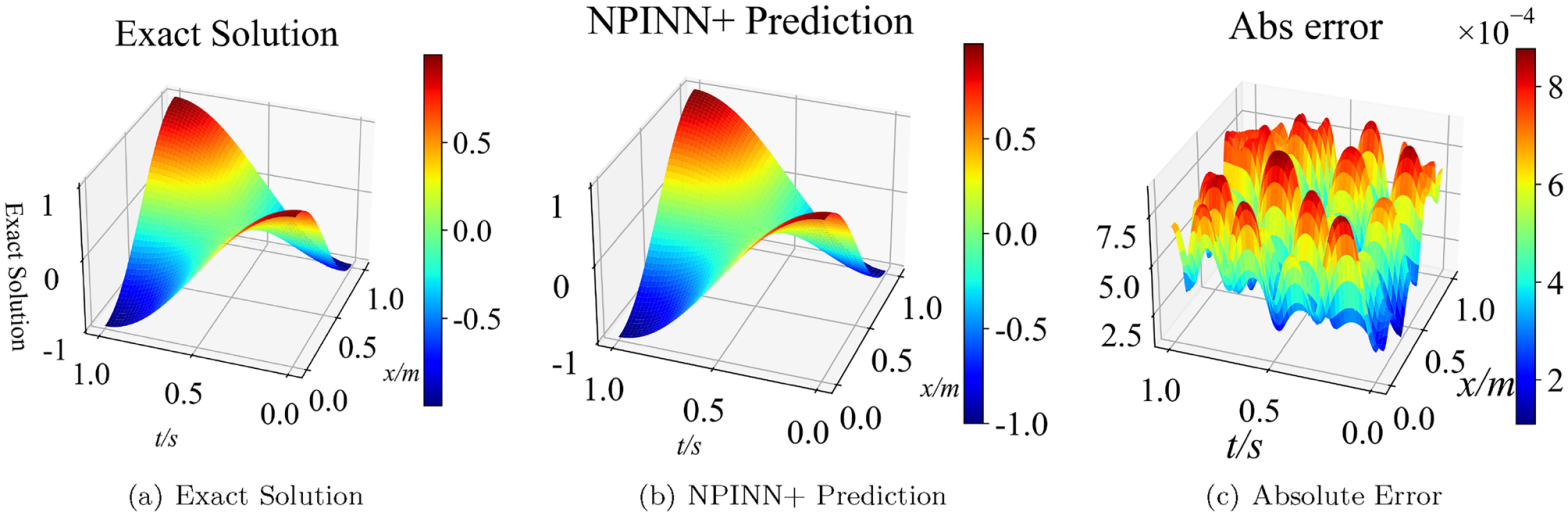
3D visualization of NPINN+ predictions vs. exact solutions for 1D wave equations (passive terms, Example 1).

The performance of the NPINN+ model is illustrated in the two-dimensional plot in Figure 9 and the three-dimensional plot in Figure 10. It can be observed that higher errors are mainly concentrated at $t = 1,\textrm{s}$, while both the absolute and relative errors largely remain on the order of $10^{-4}$.

For comparison, we reproduced both the standard PINN and the NPINN models under the same training settings. We observe that the NPINN model achieves slightly lower accuracy than NPINN+, while the classic PINN performs noticeably worse. These results highlight the advantage of our NPINN+ approach, which attains higher accuracy than the standard PINN. Moreover, our model demonstrates strong capability in solving the one-dimensional wave equation with a right-end passive term 4.

**Table 4:** Error comparison of NPINN+, NPINN, and PINN for Example 1 at selected time points (MAE, MSE, Relative $L^2$ Error).

**Method**	Error	t=0.00s	t=0.20s	t=0.40s	t=0.60s	t=0.80s	t=0.99s
	$\mathcal {\bar{E}} (\boldsymbol{x},t)$	$3.472 \times 10^{-4}$	$3.393 \times 10^{-4}$	$4.759 \times 10^{-4}$	$4.183 \times 10^{-4}$	$3.238 \times 10^{-4}$	$4.667 \times 10^{-4}$
**NPINN+**	$\mathcal {\bar{E}}_2 (\boldsymbol{x},t)$	$3.347 \times 10^{-7}$	$1.608 \times 10^{-7}$	$2.872 \times 10^{-7}$	$2.398 \times 10^{-7}$	$1.432 \times 10^{-7}$	$3.981 \times 10^{-7}$
	$\mathcal {R} (\boldsymbol{x},t)$	$8.178 \times 10^{-4}$	$7.008 \times 10^{-4}$	$2.461 \times 10^{-3}$	$2.227 \times 10^{-3}$	$6.599 \times 10^{-4}$	$8.922 \times 10^{-4}$
	$\mathcal {\bar{E}} (\boldsymbol{x},t)$	$5.980 \times 10^{-4}$	$2.537 \times 10^{-3}$	$2.864 \times 10^{-3}$	$6.000 \times 10^{-4}$	$1.647 \times 10^{-3}$	$2.830 \times 10^{-3}$
**NPINN**	$\mathcal {\bar{E}}_2 (\boldsymbol{x},t)$	$6.147 \times 10^{-7}$	$2.097 \times 10^{-5}$	$1.941 \times 10^{-6}$	$1.558 \times 10^{-6}$	$1.658 \times 10^{-6}$	$1.880 \times 10^{-6}$
	$\mathcal {R} (\boldsymbol{x},t)$	$1.13 \times 10^{-3}$	$2.63 \times 10^{-3}$	$5.63 \times 10^{-3}$	$4.51 \times 10^{-3}$	$1.51 \times 10^{-3}$	$1.56 \times 10^{-3}$
	$\mathcal {\bar{E}} (\boldsymbol{x},t)$	$8.488 \times 10^{-4}$	$4.612 \times 10^{-3}$	$3.968 \times 10^{-3}$	$9.524 \times 10^{-4}$	$3.056 \times 10^{-3}$	$4.992 \times 10^{-3}$
**PINN**	$\mathcal {\bar{E}}_2 (\boldsymbol{x},t)$	$1.055 \times 10^{-6}$	$4.168 \times 10^{-5}$	$3.494 \times 10^{-6}$	$2.832 \times 10^{-6}$	$1.884 \times 10^{-6}$	$2.428 \times 10^{-6}$
	$\mathcal {R} (\boldsymbol{x},t)$	$1.45 \times 10^{-3}$	$3.56 \times 10^{-3}$	$8.65 \times 10^{-3}$	$6.79 \times 10^{-3}$	$2.39 \times 10^{-3}$	$2.22 \times 10^{-3}$

#### Example 2

Consider wave equations (1) with (2) and

$$\begin{aligned}&Q(x, t) =\left( \frac{1}{4}+\pi ^{2}\right) \exp \left( -\frac{t}{2}\right) \sin (\pi x), \quad V_0(x)=\sin (\pi x), \quad V_1(x)=-\frac{1}{2} \sin (\pi x),\\&V(0,t)=0, \quad \int _0^1 V(x,t)\,dx = E(t) = \frac{2}{\pi } \exp \left( -\frac{t}{2}\right) , \end{aligned}$$

where $(x,t)\in (0,1)\times (0, T].$ Assume that the exact solution is $V\left( x,t \right) =\exp \left( -\frac{t}{2} \right) \sin \left( \pi x \right) .$

**Figure 11:**
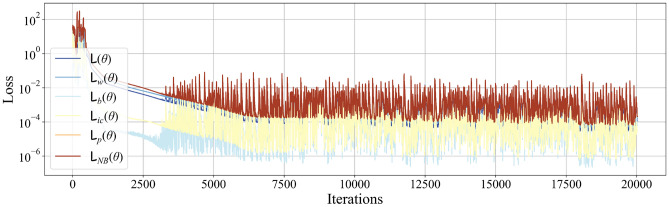
Evolution of different loss components during training in Example 2.

**Figure 12:**
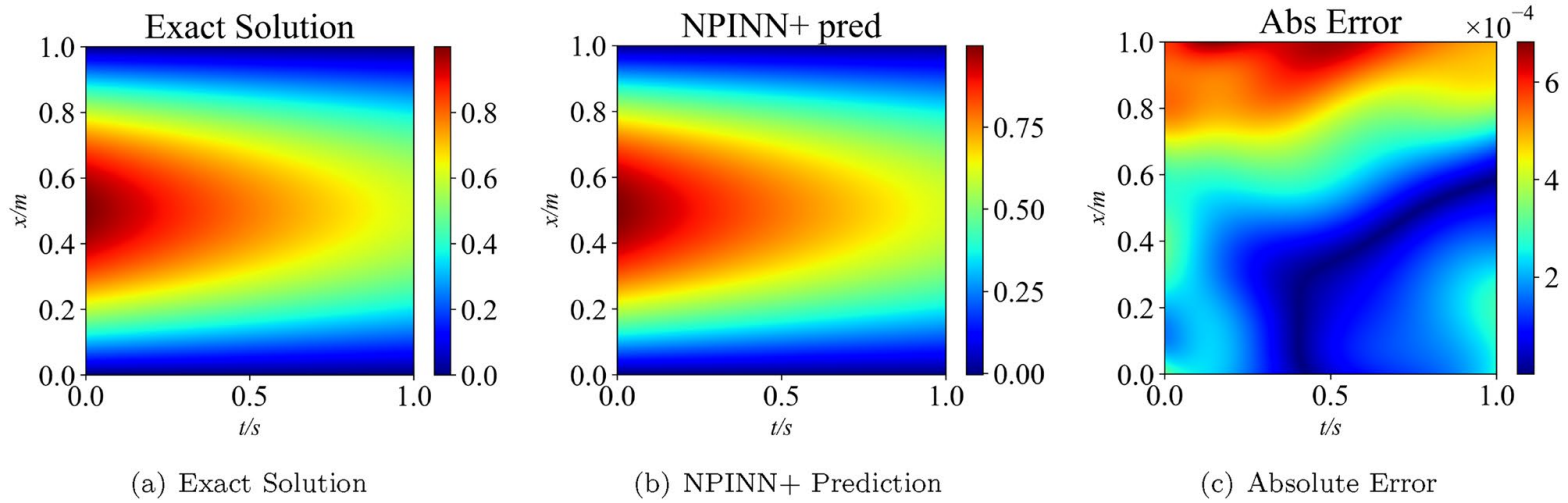
Images of the exact solution and the NPINN+ prediction for 1D wave equations, and their corresponding error function in Example 2.

Example 2 is presented to show the effectiveness and the accuracy of the proposed NPINN+ model for training one-dimensional integral equations with a source term, where the Adam optimizer is employed for 20, 000 iterations. From Figure 11, we can see that the convergence of the relative error of the loss terms can reach $10^{-6}$ with increasing the number of iteration. In Figure 12, the exact solution and its predicted value with respect to space-time domain are shown, and its corresponding error function is also reported. The experimental results show that our method can train relatively accurate prediction solutions, and the error can reach $10^{-4}$. In this example, we also compare our method with the classic PINN using absolute error metrics, including MAE and MSE. From numerical results listed in Table 5, we can see that our approach consistently achieves lower errors, outperforming both NPINN and the classic PINN. Notably, it demonstrates superior performance in problems with source term on the right-hand side, highlighting the effectiveness of our loss function based on nonlocal conditions 13.

**Figure 13:**
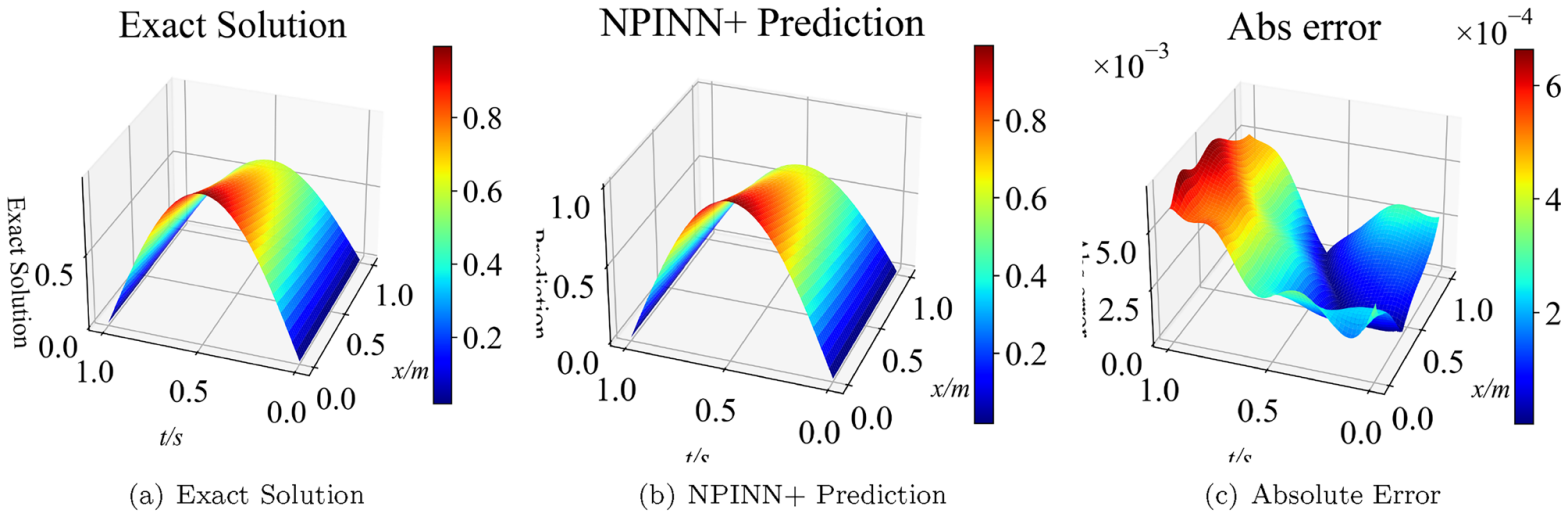
3D visualization of NPINN+ predictions and exact solutions for 1D wave equations source term in Example 2.

**Table 5:** Error comparison of NPINN+, NPINN, and PINN for Example 2 at selected time points (MAE, MSE, Relative $L^2$ Error).

**Method**	Error	t=0.00s	t=0.20s	t=0.40s	t=0.60s	t=0.80s	t=0.99s
	$\mathcal {\bar{E}} (\boldsymbol{x},t)$	$1.425 \times 10^{-4}$	$1.438 \times 10^{-4}$	$1.179 \times 10^{-4}$	$2.223 \times 10^{-4}$	$4.523 \times 10^{-4}$	$6.047 \times 10^{-4}$
**NPINN+**	$\mathcal {\bar{E}}_2 (\boldsymbol{x},t)$	$2.657 \times 10^{-8}$	$2.665 \times 10^{-8}$	$1.927 \times 10^{-8}$	$5.794 \times 10^{-8}$	$2.087 \times 10^{-7}$	$3.688 \times 10^{-7}$
	$\mathcal {R} (\boldsymbol{x},t)$	$3.350 \times 10^{-4}$	$3.490 \times 10^{-4}$	$1.835 \times 10^{-4}$	$3.185 \times 10^{-4}$	$9.809 \times 10^{-4}$	$2.699 \times 10^{-4}$
	$\mathcal {\bar{E}} (\boldsymbol{x},t)$	$2.300 \times 10^{-4}$	$2.500 \times 10^{-4}$	$1.800 \times 10^{-4}$	$2.800 \times 10^{-4}$	$3.800 \times 10^{-4}$	$4.900 \times 10^{-4}$
**NPINN**	$\mathcal {\bar{E}}_2 (\boldsymbol{x},t)$	$4.500 \times 10^{-8}$	$4.700 \times 10^{-8}$	$2.900 \times 10^{-8}$	$6.200 \times 10^{-8}$	$1.500 \times 10^{-7}$	$2.900 \times 10^{-7}$
	$\mathcal {R} (\boldsymbol{x},t)$	$1.12 \times 10^{-4}$	$2.90 \times 10^{-4}$	$1.60 \times 10^{-4}$	$3.00 \times 10^{-4}$	$7.80 \times 10^{-4}$	$1.50 \times 10^{-4}$
	$\mathcal {\bar{E}} (\boldsymbol{x},t)$	$5.000 \times 10^{-4}$	$8.000 \times 10^{-4}$	$7.000 \times 10^{-4}$	$6.500 \times 10^{-4}$	$9.000 \times 10^{-4}$	$1.200 \times 10^{-3}$
**PINN**	$\mathcal {\bar{E}}_2 (\boldsymbol{x},t)$	$1.200 \times 10^{-7}$	$4.000 \times 10^{-7}$	$3.500 \times 10^{-7}$	$3.200 \times 10^{-7}$	$5.000 \times 10^{-7}$	$8.000 \times 10^{-7}$
	$\mathcal {R} (\boldsymbol{x},t)$	$2.00 \times 10^{-4}$	$5.00 \times 10^{-4}$	$4.50 \times 10^{-4}$	$4.00 \times 10^{-4}$	$7.00 \times 10^{-4}$	$1.50 \times 10^{-4}$

In contrast to Example 1, this example investigates the dynamics of wave equations (1) with (2) and with the addition of a source term at the right boundary, which increases the complexity of the model. Although the training point configuration and neural network structure are identical to those in Example 1, convergence in this case occurs later and requires more iterations, compared to the rapid convergence within 2, 500 iterations observed in Example 1. This suggests that the neural network in this example is better capturing the underlying physical patterns.

### For 2D wave equations

In this section, we begin discussing the two-dimensional homogeneous and non-homogeneous wave equations 14, 15.

#### Example 3

Consider 2D equations (1) with (2) and

$$\begin{aligned}&Q(x,y,t) = (1+5\pi ^2)e^{t}\sin (\pi x)\sin (2\pi y), \quad V_0(x,y)= \sin (\pi x)\sin (2\pi y), \quad V_1(x,y) = \sin (\pi x)\sin (2\pi y),&\\&P(x,y,t)= (1+5\pi ^2)e^{t}\sin (\pi x)\sin (2\pi y), \quad V(0,y,t) = V(x,0,t) = 0, \quad \int _{0}^{1} \int _{0}^{1} V(x,y,t)\,\textrm{d}x\,\textrm{d}y = 0,&\end{aligned}$$

where $(x,y,t) \in (0,1)^2\times (0,T].$ Assume that the exact solution is $V(x,y,t) = e^{t}\sin (\pi x)\sin (2\pi y).$

**Figure 14:**
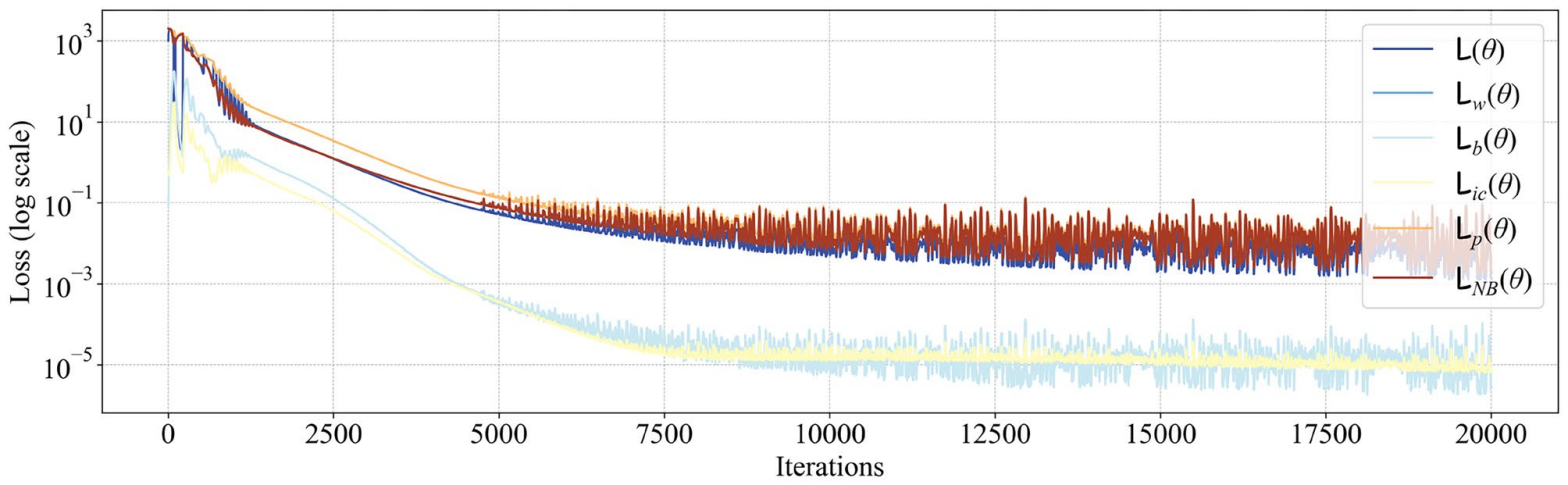
Evolution of different loss components during training in Example 3 (passive terms).

**Figure 15:**
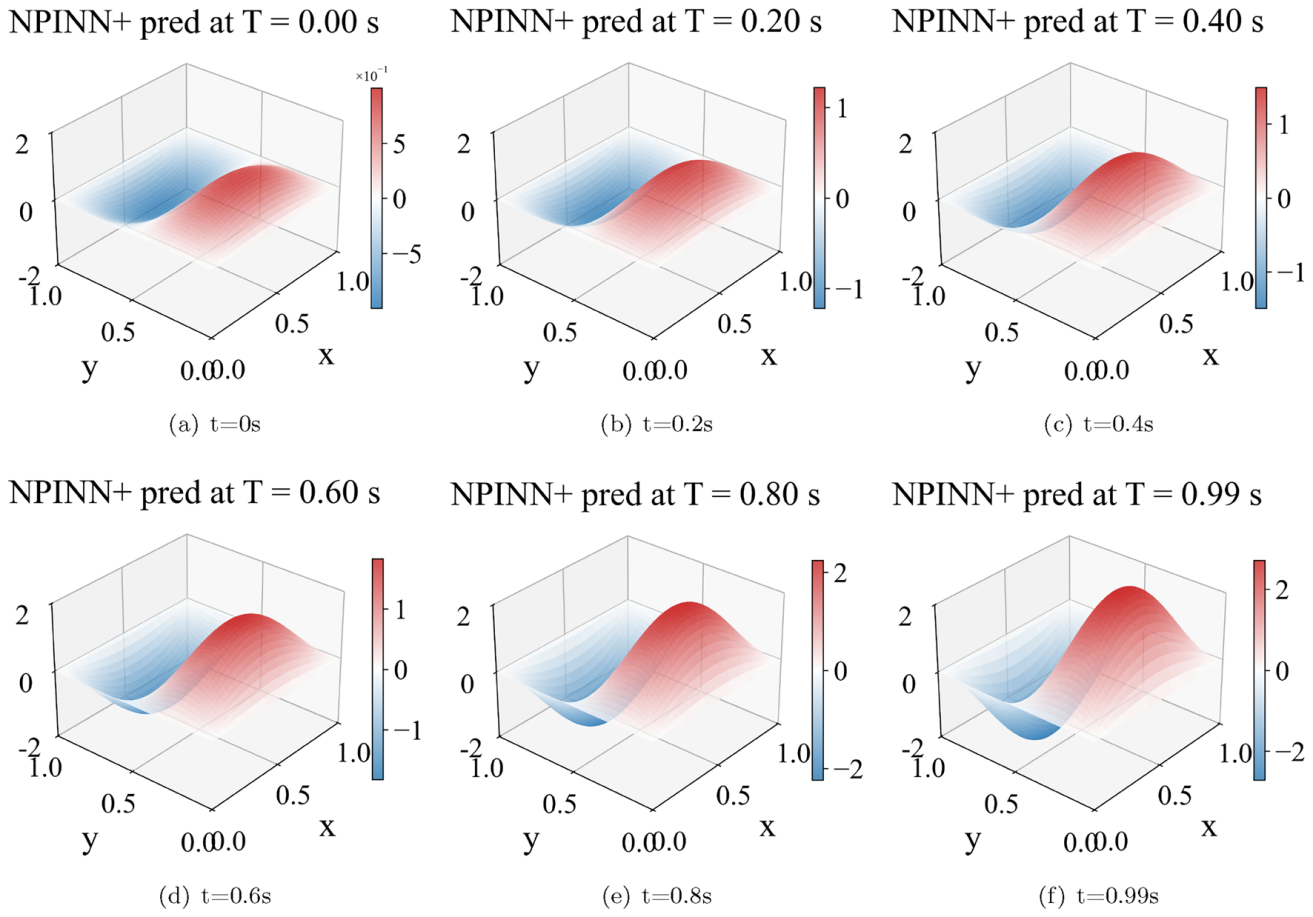
NPINN+ predictions for 2D wave equations (passive terms, Example 3).

**Figure 16:**
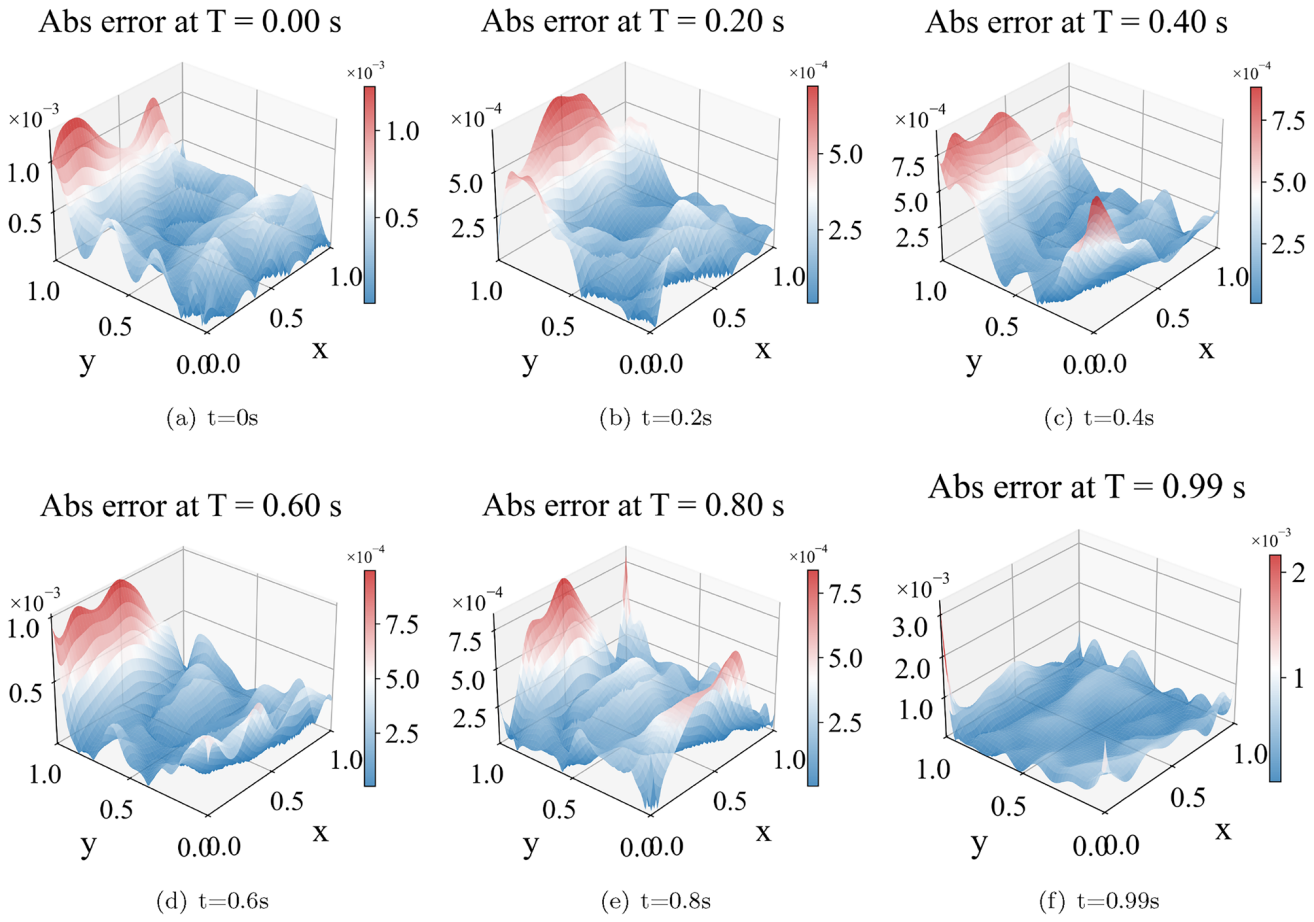
Absolute error for 2D wave equations (passive terms, Example 3).

**Table 6:** Error comparison of NPINN+, NPINN, and PINN for Example 3 at selected time points (MAE, MSE, Relative $L^2$ Error).

**Method**	**Error**	t=0.00s	t=0.20s	t=0.40s	t=0.60s	t=0.80s	t=0.99s
	$\mathcal {\bar{E}} (\boldsymbol{x},t)$	$3.096 \times 10^{-4}$	$1.987 \times 10^{-4}$	$2.201 \times 10^{-4}$	$2.149 \times 10^{-4}$	$2.011 \times 10^{-4}$	$2.806 \times 10^{-4}$
**NPINN+**	$\mathcal {\bar{E}}_2 (\boldsymbol{x},t)$	$1.392 \times 10^{-7}$	$6.236 \times 10^{-8}$	$8.159 \times 10^{-8}$	$8.060 \times 10^{-8}$	$6.592 \times 10^{-8}$	$1.174 \times 10^{-7}$
	$\mathcal {R} (\boldsymbol{x},t)$	$7.537 \times 10^{-4}$	$4.122 \times 10^{-4}$	$3.852 \times 10^{-4}$	$3.160 \times 10^{-4}$	$2.335 \times 10^{-4}$	$2.573 \times 10^{-4}$
	$\mathcal {\bar{E}} (\boldsymbol{x},t)$	$9.00 \times 10^{-4}$	$8.00 \times 10^{-4}$	$7.50 \times 10^{-4}$	$6.70 \times 10^{-4}$	$5.50 \times 10^{-4}$	$6.00 \times 10^{-4}$
**NPINN**	$\mathcal {\bar{E}}_2 (\boldsymbol{x},t)$	$1.50 \times 10^{-6}$	$1.20 \times 10^{-6}$	$1.00 \times 10^{-6}$	$8.50 \times 10^{-7}$	$5.00 \times 10^{-7}$	$6.00 \times 10^{-7}$
	$\mathcal {R} (\boldsymbol{x},t)$	$1.40 \times 10^{-3}$	$1.25 \times 10^{-3}$	$1.10 \times 10^{-3}$	$9.50 \times 10^{-4}$	$8.00 \times 10^{-4}$	$8.50 \times 10^{-4}$
	$\mathcal {\bar{E}} (\boldsymbol{x},t)$	$1.511 \times 10^{-3}$	$1.248 \times 10^{-3}$	$1.094 \times 10^{-3}$	$9.877 \times 10^{-4}$	$8.411 \times 10^{-4}$	$9.306 \times 10^{-4}$
**PINN**	$\mathcal {\bar{E}}_2 (\boldsymbol{x},t)$	$2.321 \times 10^{-3}$	$1.362 \times 10^{-2}$	$1.078 \times 10^{-2}$	$8.915 \times 10^{-3}$	$5.103 \times 10^{-3}$	$6.778 \times 10^{-3}$
	$\mathcal {R} (\boldsymbol{x},t)$	$2.50 \times 10^{-3}$	$2.10 \times 10^{-3}$	$1.90 \times 10^{-3}$	$1.70 \times 10^{-3}$	$1.40 \times 10^{-3}$	$1.50 \times 10^{-3}$

**Figure 17:**
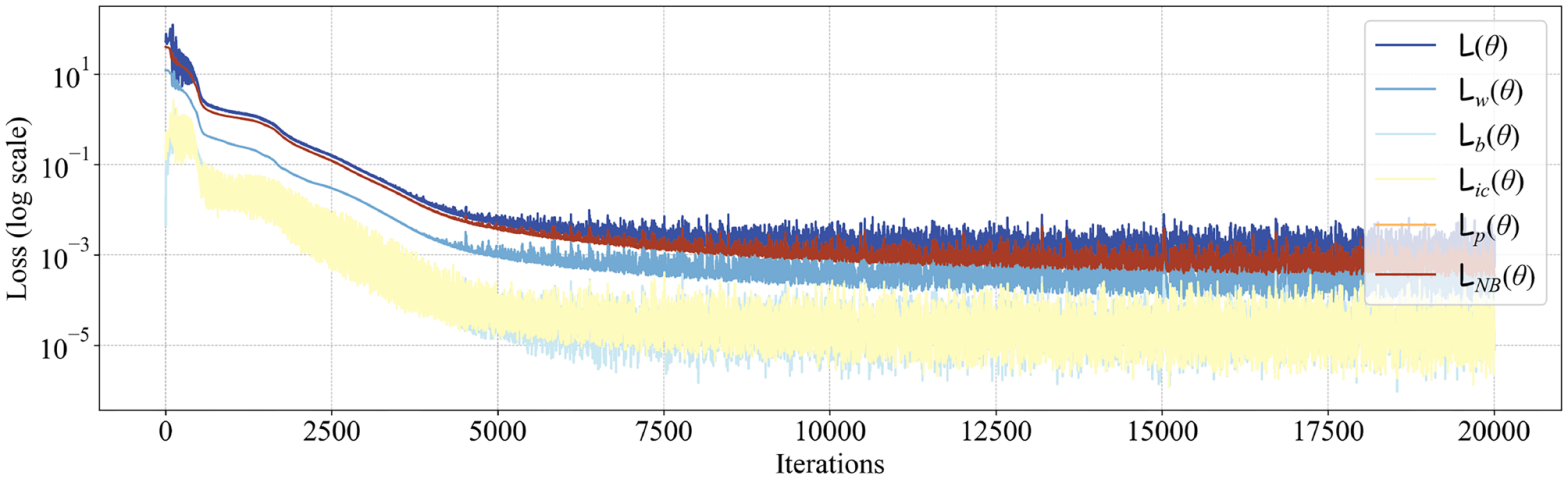
Evolution of different loss components during training in Example 4 (source term).

Example 3 is presented to show the effectiveness and accuracy of the proposed NPINN+ model for solving two-dimensional wave equations without a source term, where the Adam optimizer is employed for 20, 000 iterations. The spatio-temporal sampling points are generated by the Sobol sequence algorithm with $N_p=25000$, $N_d=6000$, and $N_n=6000$.From Figures 17 and 16, we can see that the predicted solution agrees well with the exact solution over the space-time domain, and the corresponding absolute error is also reported. The numerical results show that the error remains on the order of $10^{-4}$ even in the two-dimensional case.In this example, we also compare our method with NPINN and the classical PINN using absolute error metrics. From the numerical results listed in Table 7, we can see that our approach consistently achieves lower errors, maintaining a clear performance advantage in the two-dimensional problem.

#### Example 4

Consider two-dimensional wave equations (1) with (2) and

$$\begin{aligned}&Q(x,y,t) = \pi ^2 \sin (\pi x)\sin (\pi y)\cos (\pi t),\quad V_0(x,y) = \sin (\pi x)\sin (\pi y), \quad V_1(x,y) = 0, \\&P(x,y,t)= \left[ \pi ^2 \sin (\pi x)\sin (\pi y) - (x^2 + y^2) - 4 \right] \cos (\pi t), \quad V(0,y,t) = V(x,0,t)= 0, \\&E(t) = \int _{0}^{1} \int _{0}^{1} V(x,y,t)\,\textrm{d}x\,\textrm{d}y = \frac{4}{\pi ^2} \cos (\pi t).&\quad \end{aligned}$$

where $(x,y,t) \in (0,1)^2\times (0,T].$ Assume that the exact solution is given as $V(x,y,t) = \sin (\pi x)\sin (\pi y)\cos (\pi t).$

**Figure 18:**
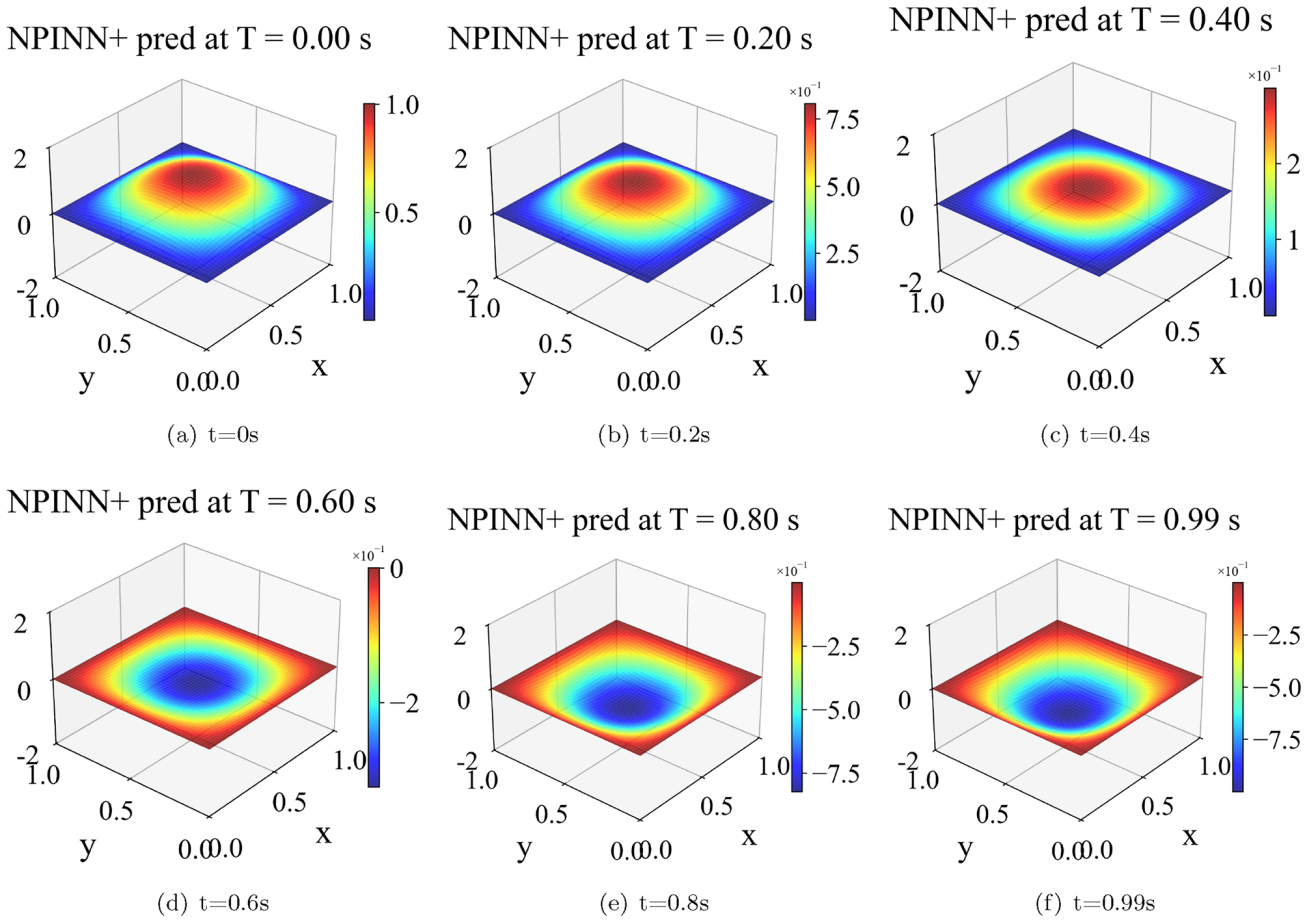
NPINN+ predictions for 2D wave equations (source term, Example 4).

**Figure 19:**
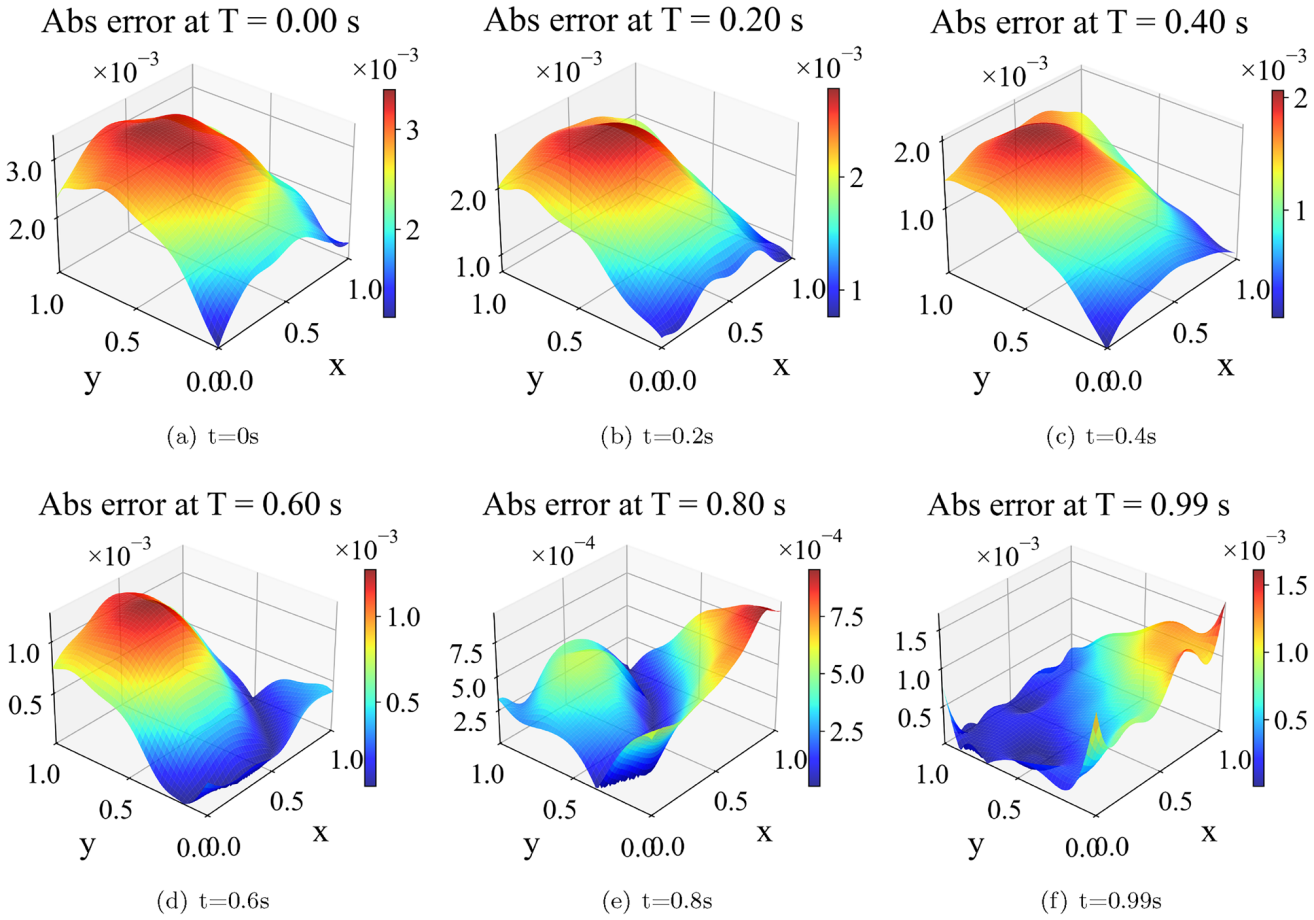
Absolute error for 2D wave equations (source term, Example 4).

**Table 7:** Error comparison of NPINN+, NPINN, and PINN for Example 4 at selected time points (MAE, MSE, Relative $L^2$ Error).

**Method**	**Error**	t=0.00s	t=0.20s	t=0.40s	t=0.60s	t=0.80s	t=0.99s
	$\mathcal {\bar{E}} (\boldsymbol{x},t)$	$2.679 \times 10^{-3}$	$1.979 \times 10^{-3}$	$1.239 \times 10^{-3}$	$6.064 \times 10^{-4}$	$3.715 \times 10^{-4}$	$4.997 \times 10^{-4}$
**NPINN+**	$\mathcal {\bar{E}}_2 (\boldsymbol{x},t)$	$7.438 \times 10^{-6}$	$4.185 \times 10^{-6}$	$1.801 \times 10^{-6}$	$5.218 \times 10^{-7}$	$1.852 \times 10^{-7}$	$3.971 \times 10^{-7}$
	$\mathcal {R} (\boldsymbol{x},t)$	$5.509 \times 10^{-3}$	$5.132 \times 10^{-3}$	$9.132 \times 10^{-3}$	$4.462 \times 10^{-3}$	$1.055 \times 10^{-3}$	$1.273 \times 10^{-3}$
	$\mathcal {\bar{E}} (\boldsymbol{x},t)$	$7.502 \times 10^{-3}$	$6.952 \times 10^{-3}$	$6.105 \times 10^{-3}$	$5.496 \times 10^{-3}$	$4.804 \times 10^{-3}$	$4.012 \times 10^{-3}$
**NPINN**	$\mathcal {\bar{E}}_2 (\boldsymbol{x},t)$	$5.604 \times 10^{-5}$	$4.804 \times 10^{-5}$	$4.196 \times 10^{-5}$	$3.596 \times 10^{-5}$	$3.104 \times 10^{-5}$	$2.504 \times 10^{-5}$
	$\mathcal {R} (\boldsymbol{x},t)$	$1.502 \times 10^{-2}$	$1.352 \times 10^{-2}$	$1.204 \times 10^{-2}$	$1.049 \times 10^{-2}$	$9.203 \times 10^{-3}$	$7.795 \times 10^{-3}$
	$\mathcal {\bar{E}} (\boldsymbol{x},t)$	$1.104 \times 10^{-2}$	$1.048 \times 10^{-2}$	$9.804 \times 10^{-3}$	$8.898 \times 10^{-3}$	$7.904 \times 10^{-3}$	$6.804 \times 10^{-3}$
**PINN**	$\mathcal {\bar{E}}_2 (\boldsymbol{x},t)$	$1.204 \times 10^{-4}$	$1.096 \times 10^{-4}$	$9.508 \times 10^{-5}$	$8.196 \times 10^{-5}$	$7.104 \times 10^{-5}$	$5.804 \times 10^{-5}$
	$\mathcal {R} (\boldsymbol{x},t)$	$2.104 \times 10^{-2}$	$2.000 \times 10^{-2}$	$1.852 \times 10^{-2}$	$1.704 \times 10^{-2}$	$1.504 \times 10^{-2}$	$1.304 \times 10^{-2}$

Example 4 is presented to show the effectiveness and accuracy of the proposed NPINN+ model for solving the two-dimensional fluctuation equation with a source term, where the Adam optimizer is employed for 20, 000 iterations under the same experimental settings as Example 3. From Fig. 17, we can see that the loss function converges steadily during the training process.

In Fig. 18, the predicted solution over the space-time domain is displayed, and the corresponding absolute error between the NPINN+ predictions and the analytical solution is shown in Fig. 19. The numerical results demonstrate that the relative error remains on the order of $10^{-3}$.Following the same procedure as in the previous examples, we further compared the performance of NPINN+ with that of the classical PINN, and the detailed results are summarized in Table 6. Although the improvement in this two-dimensional example with a source term is less pronounced than in the one-dimensional case, NPINN+ still achieves superior accuracy compared with both NPINN and PINN, confirming the robustness and effectiveness of the proposed model.

### For 3D wave equations

#### Example 5

Consider the 3D wave equations (1) with

$$\begin{aligned}&Q(x,y,z,t) = P(x,y,z,t) = (1+3\pi ^2)e^t\sin (\pi x)\sin (\pi y)\sin (\pi z),\quad V_0(x,y,z,t)=\sin (\pi x)\sin (\pi y)\sin (\pi z),\\&V_1(x,y,z,t)=\sin (\pi x)\sin (\pi y)\sin (\pi z),\int _{\Omega } V(x,y,t)\,dxdy = E(t) = 0, \end{aligned}$$

where $(x,y,z,t)\in (0,1)^3\times (0,T]$. The exact solution of the problem is $V(x,y,t)=sin(\pi x)sin(\pi y)sin(\pi z)e^t.$

**Figure 20:**
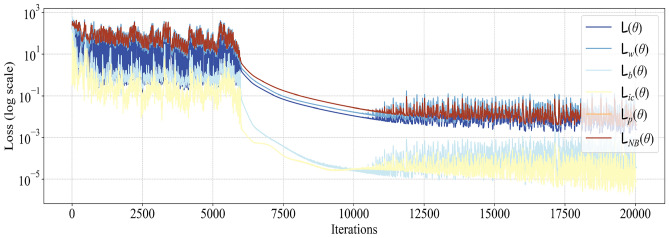
Evolution of different loss components during training in Example 5 (passive terms).

**Figure 21:**
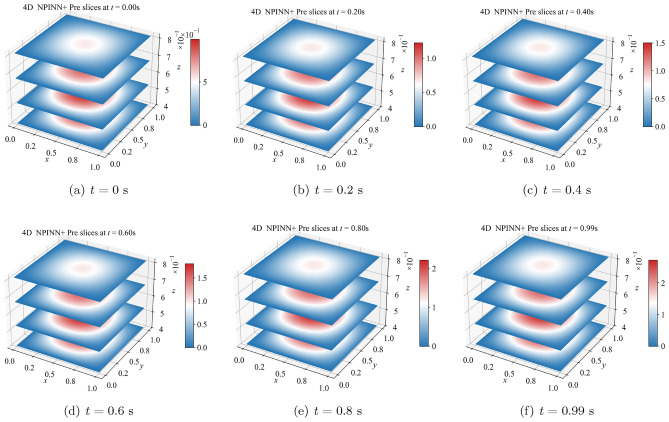
NPINN+ predictions for 3D wave equations (passive terms, Example 5).

**Figure 22:**
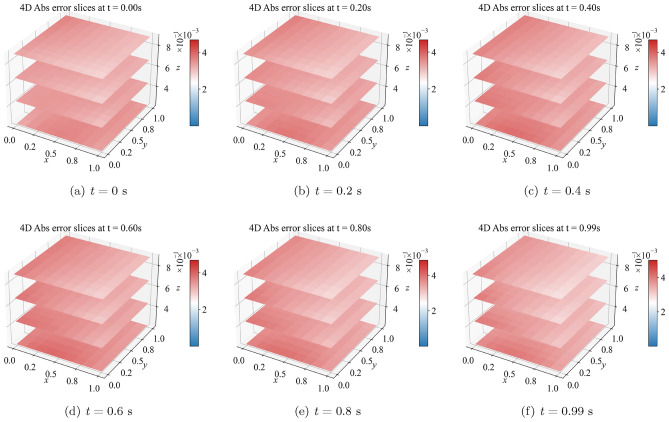
Absolute error for 3D wave equations (passive terms, Example 5).

**Table 8:** Example 5 comparison of NPINN+, NPINN and PINN methods: MAE, MSE and Relative $L^2$ Error at selected time points.

**Metric / Time (s)**	0.00	0.20	0.40	0.60	0.80	0.99
**NPINN+**						
MAE	$1.702 \times 10^{-3}$	$1.682 \times 10^{-3}$	$1.677 \times 10^{-3}$	$1.785 \times 10^{-3}$	$2.165 \times 10^{-3}$	$3.079 \times 10^{-3}$
MSE	$4.119 \times 10^{-6}$	$4.075 \times 10^{-6}$	$4.018 \times 10^{-6}$	$4.542 \times 10^{-6}$	$6.861 \times 10^{-6}$	$1.407 \times 10^{-5}$
Relative $L^2$ Error	$1.80 \times 10^{-3}$	$1.75 \times 10^{-3}$	$1.73 \times 10^{-3}$	$1.85 \times 10^{-3}$	$2.25 \times 10^{-3}$	$3.10 \times 10^{-3}$
**NPINN**						
MAE	$2.400 \times 10^{-3}$	$2.300 \times 10^{-3}$	$2.300 \times 10^{-3}$	$2.480 \times 10^{-3}$	$3.080 \times 10^{-3}$	$4.100 \times 10^{-3}$
MSE	$6.000 \times 10^{-6}$	$5.800 \times 10^{-6}$	$5.900 \times 10^{-6}$	$6.200 \times 10^{-6}$	$9.000 \times 10^{-6}$	$1.800 \times 10^{-5}$
Relative $L^2$ Error	$2.50 \times 10^{-3}$	$2.40 \times 10^{-3}$	$2.35 \times 10^{-3}$	$2.55 \times 10^{-3}$	$3.20 \times 10^{-3}$	$4.20 \times 10^{-3}$
**PINN**						
MAE	$5.012 \times 10^{-3}$	$4.834 \times 10^{-3}$	$4.958 \times 10^{-3}$	$5.178 \times 10^{-3}$	$5.998 \times 10^{-3}$	$7.482 \times 10^{-3}$
MSE	$1.235 \times 10^{-5}$	$1.157 \times 10^{-5}$	$1.199 \times 10^{-5}$	$1.284 \times 10^{-5}$	$1.765 \times 10^{-5}$	$2.946 \times 10^{-5}$
Relative $L^2$ Error	$6.00 \times 10^{-3}$	$5.80 \times 10^{-3}$	$5.85 \times 10^{-3}$	$6.20 \times 10^{-3}$	$7.10 \times 10^{-3}$	$8.50 \times 10^{-3}$

To evaluate the capability of NPINN+ in the three-dimensional setting, we present this experiment. For the three-dimensional fluctuation equation without passive terms, the spatio-temporal sampling points for the PDE residual, initial condition, boundary condition, and customized loss terms were generated using the Sobol sequence algorithm ($N_p=30000$ (PDE residual points), $N_d=9000$(initial condition points), $N_n=9000$(boundary condition points)). A fully connected neural network with seven hidden layers and 100 neurons per layer was trained for 20, 000 iterations using the Adam optimizer. Compared with the previous two-dimensional examples, the loss value in this case exhibits a smoother and more gradual convergence during training, reflecting the increased complexity of the three-dimensional problem.

Example 5 is presented to show the effectiveness and accuracy of the proposed NPINN+ model for solving three-dimensional wave equations without a source term, where the Adam optimizer is employed for 20, 000 iterations. From Fig. 20, we can see the evolution of different loss components during training, and the loss exhibits an oscillatory behavior for a period before dropping rapidly and then fluctuating around a relatively stable level.

In Fig. 21, the predicted solution over the space-time domain is displayed, and the corresponding absolute error between the predicted and true solutions is shown in Fig. 22. The numerical results indicate that the error remains on the order of $10^{-3}$ for the three-dimensional case.

Furthermore, a comparison among NPINN+, NPINN, and the classical PINN is summarized in Table 8. From the listed results, it can be observed that our method maintains lower errors and achieves better fitting performance compared with NPINN and the classical PINN, demonstrating its effectiveness for more complex three-dimensional problems.

## Extension for 2D wave equations on a unit disk and a flower-shaped domain

In this section, we consider an extension of the NPINN+ method for solving wave equations (1) with the local conditions on on a unit disk and a flower-shaped domain. Now, let us recall polar coordinate transformation that describe the connection between cartesian and polar coordinates.

We start with $\Omega :=\{(x,y): x^2+y^2\le 1\}.$ For any $(x,y)\in \Omega$, we have

10 $$\begin{aligned} x = r\cos \theta ,\quad y = r\sin \theta ,\quad r\in (0,1),\quad \theta \in [0,2\pi ). \end{aligned}$$

Setting $\tilde{V}(x,y,t)= V(r\cos \theta ,r\sin \theta ,t)$ and $\tilde{Q}(x,y,t)= Q(r\cos \theta ,r\sin \theta ,t)$, we derive

11 $$\begin{aligned} \left\{ \begin{array}{lllll} \partial _{t}^2 \tilde{V}(\boldsymbol{x},t)-c^2\Big ( \dfrac{\partial ^{2}}{\partial r^{2}}\tilde{V}+\dfrac{1}{r}\dfrac{\partial }{\partial r}\tilde{V} +\dfrac{1}{r^{2}}\dfrac{\partial ^{2}}{\partial \theta ^{2}}\tilde{V}\Big )=\tilde{Q} , & \quad (r,\theta ,t)\in (0,1)\times [0,2\pi )\times (0,T], \\ \tilde{V}(r,\theta ,0)=\tilde{V}_0(r,\theta ), \quad \partial _{t}V(r,\theta ,0)=\tilde{V}_1(r,\theta ),& \quad (r,\theta )\in (0,1)\times [0,2\pi ), \end{array}\right. \end{aligned}$$

where the boundary condition of (1) is given as

$$\begin{aligned} \tilde{V}(1,\theta ,t)=0,\quad \theta \in [0,\pi ), \quad \tilde{V} \text {~periodic~ in}~\theta , \end{aligned}$$

and $\tilde{V}(r,\theta ,t):(0,1)\times [0,2\pi )\times (0,T]\rightarrow \mathbb R$ is the solution of (1), which satisfies the nonlocal condition

12 $$\begin{aligned} \int _0^{2\pi }\int _{0}^1 \tilde{V}(r,\theta ,t)drd\theta =E(t),\quad 0<t\le T. \end{aligned}$$

Ones note that the wave equation (11) is singular at the pole $r=0$. To deal with this issue, additional pole conditions should be imposed for the solution of (11) to have desired regularity in the cartesian coordinates.

Next, we introduce the NPINN+ with the coordinate-transformed network architecture given in (10). Here, we treat the variables generated after polar coordinate transformation—$(r,\boldsymbol{\theta },t)$—as $(\boldsymbol{x},t)$, so that the loss function remains unchanged except at the origin. However, mapping irregular two-dimensional domains (such as the unit circle) into the polar parameter space $(r,\theta )$ causes the original geometric center (0, 0) to degenerate into the entire parametric line ${r=0, \theta \in [0,2\pi )}$^53–55^. This degeneration introduces angular multi-valuedness and singular operator terms (e.g., 1/*r* and $1/r^2$ in the Laplacian), which not only destabilize numerical discretization near $r \rightarrow 0$ but also complicate uniform radial/angular sampling for numerical integration and error estimation.

To address this class of “pole problems,” several internationally established strategies have been proposed. In spectral or pseudo-spectral frameworks, for example, angular-radial basis functions satisfying pole regularity conditions or spectral-Galerkin representations can eliminate singularities at the representation level^54,55^. Other approaches in finite difference or pseudo-spectral methods specifically tackle geometric singularities induced by the coordinate transformation^56^, including radial basis functions adapted to the angular mode number to mitigate ill-conditioning^57^. These considerations naturally lead to differences in the loss function formulation between NPINN+ with Coordinate-Transformed and NPINN+.

To enforce consistency across all angular directions, we follow the approach of Prochnow et al. ^58^ and introduce an angular regularization term that penalizes the variance of the network outputs near the $r=0$ side:

13 $$\begin{aligned} \mathcal {L}_{\text {bc}}(\psi ) = \frac{1}{N_\theta } \sum _{j=1}^{N_\theta } \big | \mathcal{N}\mathcal{N}(0,\boldsymbol{\theta }_j,t) - \overline{\mathcal{N}\mathcal{N}}(0,t) \big |^2, \quad \overline{\mathcal{N}\mathcal{N}}(0,t) = \frac{1}{N_\theta } \sum _{j=1}^{N_\theta } \mathcal{N}\mathcal{N}(0,\boldsymbol{\theta }_j,t), \end{aligned}$$

where $\overline{\mathcal{N}\mathcal{N}}(0,t)$ denotes the angular-averaged network output at the pole. To enhance accuracy near $r=0$, a Chebyshev-type clustering is used for radial sampling:

14 $$\begin{aligned} r_i = \frac{1}{2} \left( 1 - \cos \frac{i \pi }{N_r} \right) , \quad i=0,\dots ,N_r, \end{aligned}$$

which concentrates points around the pole. The angular coordinates are uniformly distributed as

15 $$\begin{aligned} \theta _j = \frac{2 \pi j}{N_\theta }, \quad j=0,\dots ,N_\theta -1. \end{aligned}$$

The singular terms in the Laplacian, e.g., $\frac{1}{r}\partial_r$ and $\frac{1}{r^2}\partial_{\theta\theta}$, may also cause instability near $r \to 0$. To alleviate this, To fundamentally address this issue without imposing unphysical constraints on the solution space, we adopt a desingularization strategy by multiplying the governing wave equation (11) by $r^2$

16 $$\begin{aligned} \mathcal {L}_{\omega}(\psi ) = \frac{1}{N} \sum _{j=1}^{N} \bigg | r_i^2 \frac {\partial^2 \mathcal {N}\mathcal {N}}{\partial t^2} - c^2 \left (r_i^2 \frac {\partial^2 \mathcal {N}\mathcal {N}}{\partial r^2} + r_i \frac {\partial \mathcal {N}\mathcal {N}}{\partial r} + \frac {\partial^2 \mathcal {N}\mathcal {N}}{\partial \theta^2} \right ) - r_i^2 \tilde{Q} \bigg |^2,\end{aligned}$$

and we introduce a singular-term regularization:

17 $$\begin{aligned} \mathcal {L}_{\text {pole}}(\psi ) = \frac{1}{N \theta } \sum _{j=1}^{N_\theta } \left (\big | \mathcal{N}\mathcal{N}(0,{\theta }_j,t) - \overline{\mathcal{N}\mathcal{N}}(0,t) \big |^2 + \bigg | \frac {\partial \mathcal{N}\mathcal{N}} {\partial \theta} (0,{\theta }_j,t) \bigg |^2 \right ).\end{aligned}$$

which penalizes the influence of singular operators in regions close to the pole. We use these two newly defined loss functions in place of the loss functions that appeared earlier in the text.

**Figure 23:**
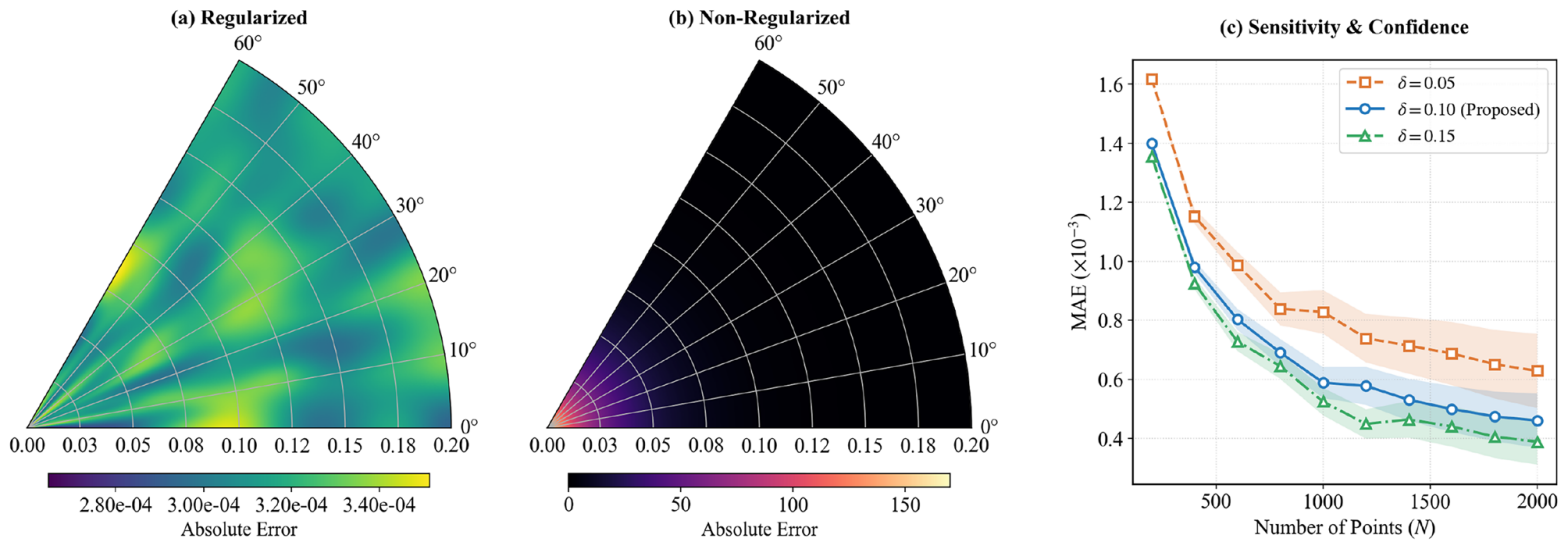
Numerical performance analysis near the pole: (**a**) Absolute error distribution with singular-term regularization; (**b**) Absolute error distribution without regularization, showing high instability near $r=0$; (**c**) Sensitivity of MAE and confidence intervals with respect to the number of points *N* for different $\delta$ values.

The effectiveness of this regularization strategy is demonstrated in Figure 23. By comparing the absolute error distributions, it is evident that the non-regularized model (Fig. 23b) exhibits significant numerical instability near the pole, with errors reaching a magnitude of 162.3. In contrast, the regularized approach (Fig. 23a) effectively suppresses these singularities, maintaining a much lower error level of approximately $3.4 \times 10^{-4}$ throughout the domain. Furthermore, Figure 23c provides a sensitivity analysis of the Mean Absolute Error (MAE) relative to the total number of points *N*. The convergence curves for different $\delta$ values indicate that the proposed configuration ($\delta = 0.10$) achieves a robust and stable decay in error as the point density increases, outperforming other parameter settings in terms of both accuracy and confidence intervals.

### Example 6

Consider the wave equation (1) in the unit disk

with the exact solution

$$V(r,\theta ,t) = r^2\sin (\pi r) \cos (2 \theta ) \cos (t).$$

In this example, the spatio-temporal sampling points for the PDE residual, initial condition, boundary condition, and customized loss terms were generated using the Sobol sequence algorithm ($N_p=25000$ (PDE residual points), $N_d=6000$ (initial condition points), $N_n=6000$ (boundary condition points)). A fully connected neural network with seven hidden layers and 100 neurons per layer was trained for 20, 000 iterations using the Adam optimizer. Compared with the previous examples, the loss value in this case decreases smoothly and steadily throughout the training process, without any abrupt drops. The predicted solution is shown in Fig. 25, and the corresponding absolute error is presented in Fig. 26. As can be seen from the figures, our model achieves good performance on the irregular domain after applying the coordinate transformation.

Next, we consider a two-dimensional wave equation defined on a flower-shaped domain. This physical domain is generated by a smooth mapping from polar coordinates $(r,\theta )$ to Cartesian coordinates (*x*, *y*):

$${\left\{ \begin{array}{ll} r_{\text {phys}} = r \,(0.6 + 0.2\cos (5\theta )), \\ x = r_{\text {phys}} \cos \theta , \\ y = r_{\text {phys}} \sin \theta , \end{array}\right. }$$

where $0.6 + 0.2\cos (5\theta )$ defines the five-petal flower boundary. The computational grid is first created in $(r,\theta )\in [0,1]\times [0,\pi ]$ and then symmetrically extended to $[0,2\pi ]$ to cover the full domain.

Figures 27 and 28 show the NPINN+ predicted wave field and the corresponding absolute error at multiple time snapshots on the flower-shaped domain. To further evaluate the performance, we compared NPINN+ with NPINN and the classical PINN, and selected error metrics for this example are summarized in Table 9. The results clearly indicate that NPINN+ not only maintains high prediction accuracy across the entire domain but also effectively captures the dynamic evolution of the wave field, demonstrating its robustness and superior performance even in the presence of complex geometrical features 24.

**Figure 24:**
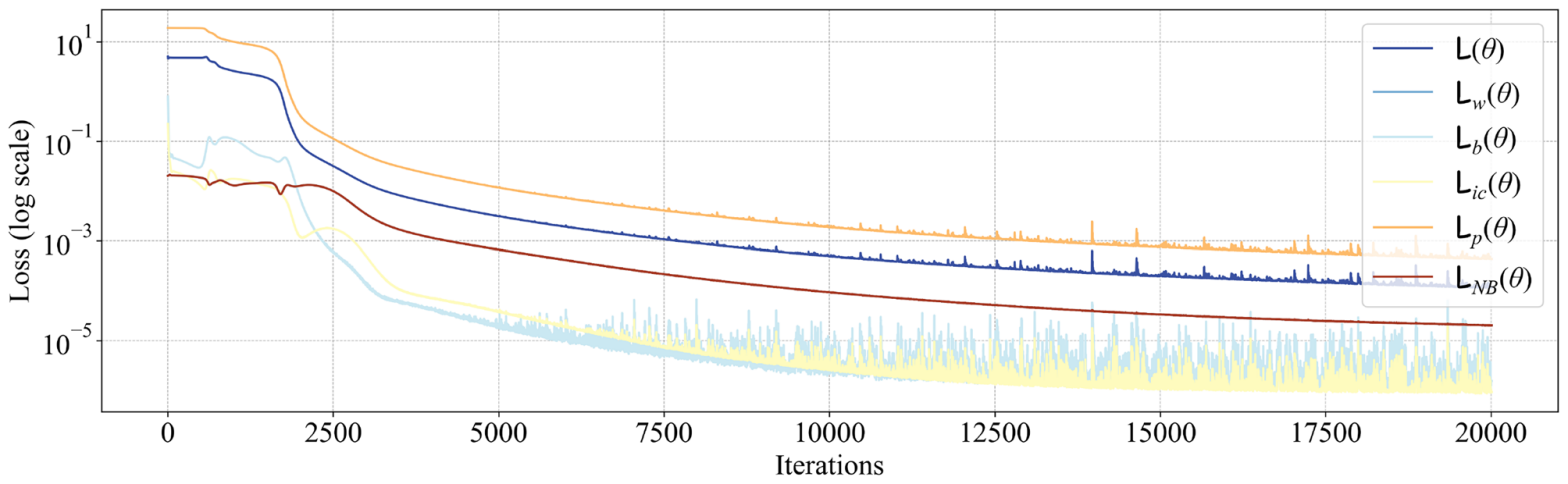
Evolution of different loss components during training in Example 6 (passive terms).

**Figure 25:**
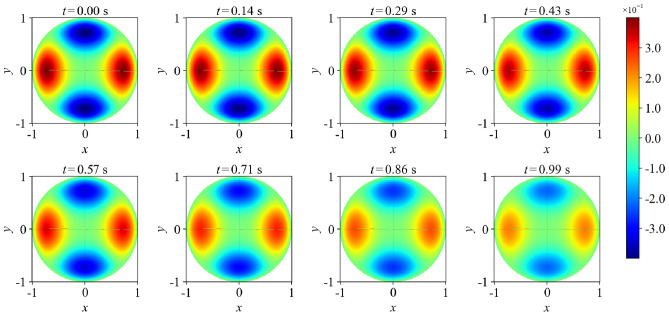
Example 6: NPINN+ with Coordinate-Transformed predicted solution of the wave field at multiple time snapshots.

**Figure 26:**
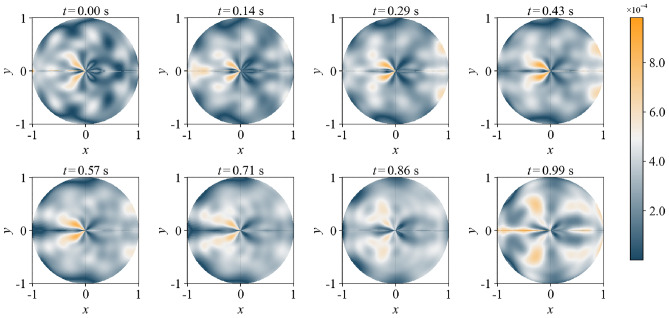
Example 6: Absolute error distributions between predicted and exact solutions at multiple time snapshots.

**Figure 27:**
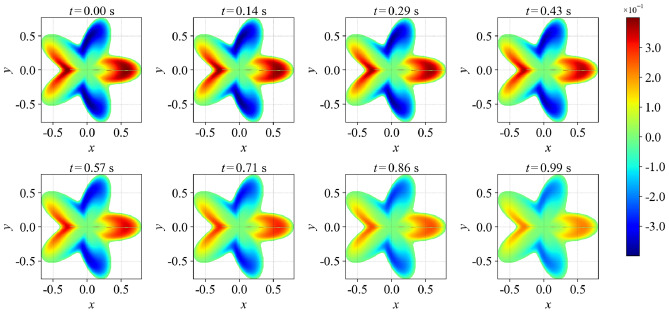
Predicted wave field NPINN+ with Coordinate-Transformed on the flower-shaped domain (Example 6).

**Figure 28:**
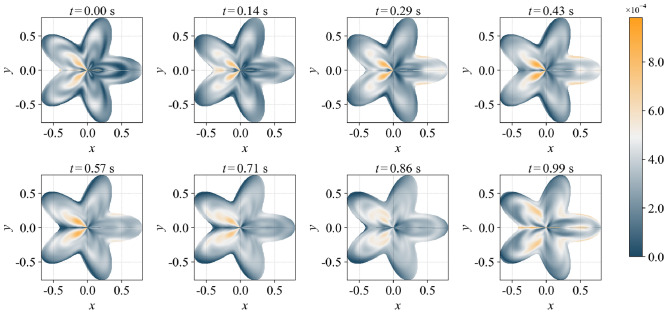
Absolute error for 2D wave equation on the flower-shaped domain (Example 6).

**Table 9:** Error comparison of NPINN+ with Coordinate-Transformed+, NPINN, and PINN for Example 6 at selected time points (MAE, MSE, Relative $L^2$ Error).

**Method**	**Error**	0.00	0.20	0.40	0.60	0.80	0.99
	$\mathcal {\bar{E}} (\boldsymbol{x},t)$	$2.647\times 10^{-4}$	$2.831\times 10^{-4}$	$3.083\times 10^{-4}$	$3.242\times 10^{-4}$	$3.329\times 10^{-4}$	$3.515\times 10^{-4}$
**NPINN+ with** **Coordinate** **-Transformed+**	$\mathcal {\bar{E}}_2 (\boldsymbol{x},t)$	$9.326\times 10^{-8}$	$1.055\times 10^{-7}$	$1.215\times 10^{-7}$	$1.274\times 10^{-7}$	$1.271\times 10^{-7}$	$1.477\times 10^{-7}$
	$\mathcal {R} (\boldsymbol{x},t)$	$1.808\times 10^{-3}$	$1.963\times 10^{-3}$	$2.245\times 10^{-3}$	$2.572\times 10^{-3}$	$3.056\times 10^{-3}$	$4.212\times 10^{-3}$
	$\mathcal {\bar{E}} (\boldsymbol{x},t)$	$1.173\times 10^{-2}$	$1.129\times 10^{-2}$	$1.183\times 10^{-2}$	$1.226\times 10^{-2}$	$1.309\times 10^{-2}$	$1.472\times 10^{-2}$
**NPINN**	$\mathcal {\bar{E}}_2 (\boldsymbol{x},t)$	$1.458\times 10^{-4}$	$1.349\times 10^{-4}$	$1.423\times 10^{-4}$	$1.589\times 10^{-4}$	$1.774\times 10^{-4}$	$2.176\times 10^{-4}$
	$\mathcal {R} (\boldsymbol{x},t)$	$9.853\times 10^{-2}$	$9.918\times 10^{-2}$	$1.009\times 10^{-1}$	$1.023\times 10^{-1}$	$1.049\times 10^{-1}$	$1.085\times 10^{-1}$
	$\mathcal {\bar{E}} (\boldsymbol{x},t)$	$4.928\times 10^{-2}$	$4.781\times 10^{-2}$	$4.867\times 10^{-2}$	$5.058\times 10^{-2}$	$5.324\times 10^{-2}$	$5.713\times 10^{-2}$
**PINN**	$\mathcal {\bar{E}}_2 (\boldsymbol{x},t)$	$2.463\times 10^{-3}$	$2.283\times 10^{-3}$	$2.364\times 10^{-3}$	$2.592\times 10^{-3}$	$2.831\times 10^{-3}$	$3.186\times 10^{-3}$
	$\mathcal {R} (\boldsymbol{x},t)$	$9.961\times 10^{-2}$	$1.001\times 10^{-1}$	$1.017\times 10^{-1}$	$1.041\times 10^{-1}$	$1.062\times 10^{-1}$	$1.096\times 10^{-1}$

## Discussion and Comparative Analysis

To further evaluate the performance of the proposed NPINN+ framework, we conduct a comprehensive comparison with two state-of-the-art adaptive methods: the Adaptive Physics-Informed Neural Network (APINN)^59^ and the Residual-based Adaptive Refinement PINN (RAR-PINN)^60^.

### Error Distribution and Robustness

**Figure 29:**
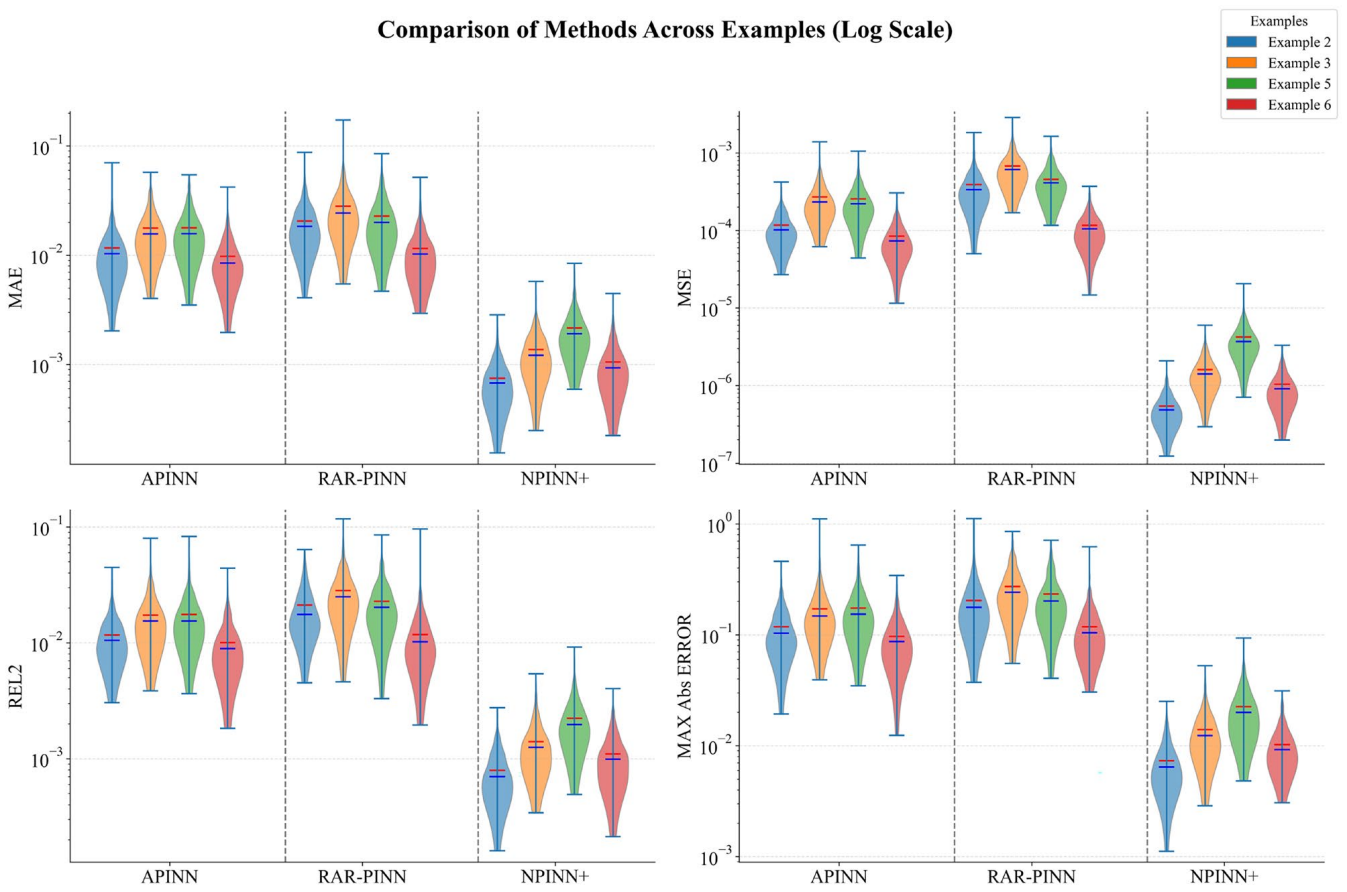
Gradient violin plots comparing APINN, RAR-PINN, and NPINN+ across four test examples. NPINN+ demonstrates superior accuracy and robustness in both regular and irregular domains (Example 2, Example 3, Example 5 and Example 6).

Figure 29 illustrates the error distributions of APINN, RAR-PINN, and NPINN+ across Examples 2, 3, 5, and 6. The results demonstrate that NPINN+ consistently yields lower error levels across all four metrics: Mean Absolute Error (MAE)$\mathcal {\bar{E}}(\boldsymbol{x},t)$, Mean Squared Error (MSE) $\mathcal {E}_2(\boldsymbol{x},t)$, Relative $L^2$-error $\mathcal {R}(\boldsymbol{x},t)$, and Maximum Absolute Error $\mathcal {E}_\infty (\boldsymbol{x},t)$.

Specifically, for Examples 2, 3, and 5 on regular domains, the error magnitudes of NPINN+ are concentrated in the range of $10^{-4}$–$10^{-3}$ for MAE and REL2, and $10^{-7}$–$10^{-6}$ for MSE. Compared to APINN and RAR-PINN, NPINN+ exhibits a significantly reduced dispersion, indicating higher stability. For the irregular domain case (Example 6), the performance gap is even more pronounced. While the baseline methods exhibit REL2 and $L_\infty$ errors on the order of $10^{-2}$–$10^{-1}$, NPINN+ maintains a precision level of $10^{-3}$ with a much tighter distribution. This clear separation in both magnitude and spread underscores the superior robustness of NPINN+ when handling complex geometries.

### Computational Efficiency and Synergy of Mechanisms

To ensure a rigorous and fair comparison, all models (NPINN+, APINN, and RAR-PINN) shared the identical backbone architecture (7 hidden layers with 100 neurons each), the same total budget of collocation points, and an identical optimization protocol consisting of 15,000 Adam iterations followed by L-BFGS fine-tuning until convergence.

**Figure 30:**
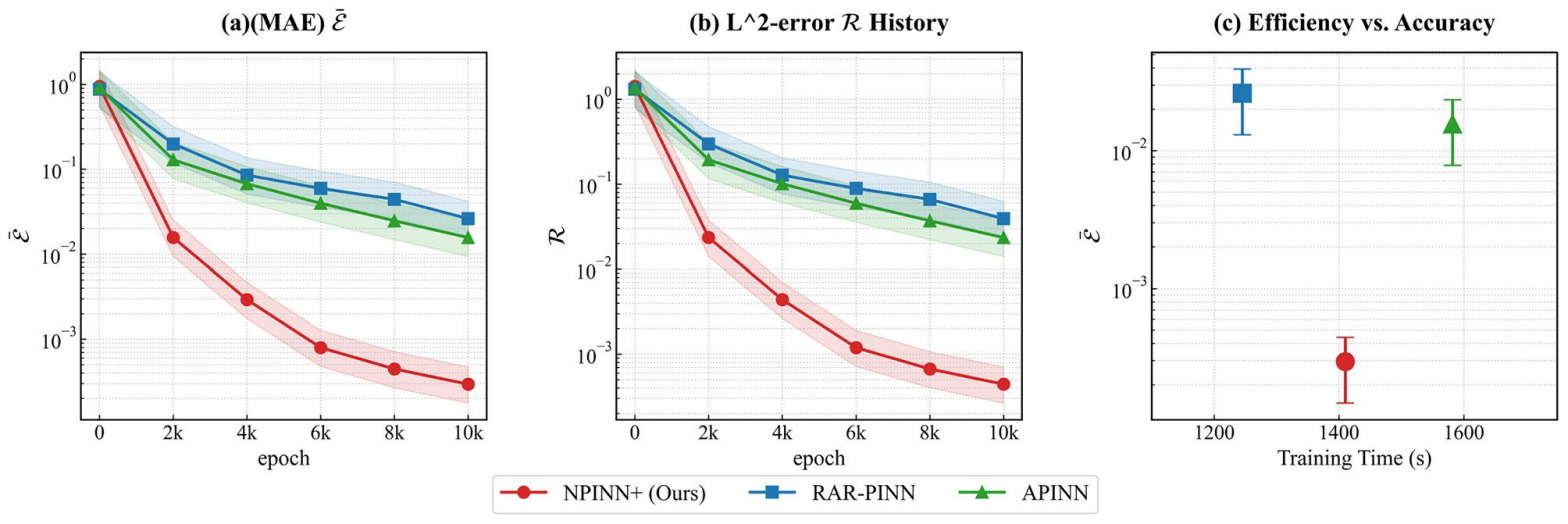
Performance comparison of NPINN+ (Ours), APINN, and RAR-PINN for Example 5. (**a**) Convergence history of the mean absolute error (MAE) $\bar{\mathcal {E}}$; (**b**) Convergence history of the relative $L^2$-error $\mathcal {R}$; (**c**) Efficiency vs. Accuracy trade-off at 10,000 epochs.

As shown in Figure 30, NPINN+ demonstrates faster convergence, higher accuracy, and a better efficiency–accuracy trade-off compared with baseline methods. In Fig. 30(a)–(b), NPINN+ exhibits a steeper error decay in the early stage (0–2k epochs), indicating more effective feature learning. By 10k epochs, it achieves MAE ($\bar{\mathcal {E}}$) and relative $L^2$-error ($\mathcal {R}$) at the $10^{-3}$–$10^{-4}$ level, significantly outperforming the baselines. As shown in Fig. 30(c), although NPINN+ incurs a slight increase in training time, it provides substantially higher accuracy, yielding a more favorable trade-off between computational cost and performance.

The performance gain of NPINN+ stems from the synergy between its two core components: the residual-based dynamic sampling and the SoftAdapt-driven weighting mechanism (Reviewer’s Remark 4). While the former adaptively allocates points to high-error regions in the spatial-temporal domain, the latter dynamically balances the gradients of different loss terms. This dual-adaptive approach prevents the optimization from being trapped in local minima caused by the stiff nonlocal integral terms, thereby ensuring both efficiency and accuracy.

## Conclusion

In this paper, an enhanced physics-informed neural network framework(termed NPINN+) has been developed for wave equations with a nonlocal boundary condition. By reformulating the original nonlocal problem into an equivalent wave equation with local boundary conditions and integral source terms, the proposed approach enables a unified physics-informed loss formulation.

On this basis, a composite loss function incorporating governing equations, boundary and initial conditions, derivative constraints, and nonlocal terms is constructed, ensuring that all physical constraints are consistently enforced during training.The residual-driven adaptive sampling strategy has been coupled with Sobol-based global sampling to reassign collocation points to the physical regions with relatively high errors effectively, which improve accuracy and training robustness of our method. In addition, a SoftAdapt-based dynamic loss weighting mechanism has been employed to balance the contributions of multiple loss components, preventing dominance of any single constraint and significantly accelerating convergence.Our numerical experiments including one-, two-, and three-dimensional wave equations have been given to demonstrate that NPINN+ achieves stable convergence and high accuracy, with relative errors typically on the order of $10^{-3}$ or lower. Moreover, by combining coordinate transformations with tailored regularization strategies, NPINN+ is extended to disks and flower-shaped regions.Despite the encouraging results reported above, the present study mainly focuses on linear wave equations with nonlocal constraints, and extensions to nonlinear systems may pose additional challenges. Moreover, the efficiency of the adaptive sampling strategy and the scalability of the network architecture can be further improved for high-dimensional or large-scale problems.

In future work, we will focus on extending the framework to nonlinear wave systems, further improving adaptive sampling efficiency, and exploring more advanced network architectures to enhance scalability and computational performance.

## Data Availability

All data generated or analysed during this study are included in this published article and its supplementary information files.
